# Identification of a robust biomarker LAPTM4A for glioma based on comprehensive computational biology and experimental verification

**DOI:** 10.18632/aging.205736

**Published:** 2024-04-12

**Authors:** Yongqi Ding, Yike Jiang, Hong Zeng, Minqin Zhou, Xuanrui Zhou, Zichuan Yu, Jingying Pan, Xitong Geng, Yanting Zhu, Hao Zheng, Shuhan Huang, Yiyang Gong, Huabin Huang, Chengfeng Xiong, Da Huang

**Affiliations:** 1 Department of Thyroid Surgery, Second Affiliated Hospital of Nanchang University, Nanchang, Jiangxi 330006, China; 2 Second College of Clinical Medicine, Nanchang University, Nanchang, Jiangxi 330006, China; 3 Department of Radiology, Second Affiliated Hospital of Nanchang University, Nanchang, Jiangxi 330006, China

**Keywords:** LAPTM4A, glioma, prognostic biomarker, immune infiltration, ceRNA

## Abstract

Background: Glioma, a highly invasive and deadly form of human neoplasm, presents a pressing need for the exploration of potential therapeutic targets. While the lysosomal protein transmembrane 4A (LATPM4A) has been identified as a risk factor in pancreatic cancer patients, its role in glioma remains unexplored.

Methods: The analysis of differentially expressed genes (DEG) was conducted from The Cancer Genome Atlas (TCGA) glioma dataset and the Genotype Tissue Expression (GTEx) dataset. Through weighted gene co-expression network analysis (WGCNA), the key glioma-related genes were identified. Among these, by using Kaplan-Meier (KM) analysis and univariate/multivariate COX methods, LAPTM4A emerged as the most influential gene. Moreover, the bioinformatics methods and experimental verification were employed to analyze its relationships with diagnosis, clinical parameters, epithelial-mesenchymal transition (EMT), metastasis, immune cell infiltration, immunotherapy, drug sensitivity, and ceRNA network.

Results: Our findings revealed that LAPTM4A was up-regulated in gliomas and was associated with clinicopathological features, leading to poor prognosis. Furthermore, functional enrichment analysis demonstrated that LATPM4A played a role in the immune system and cancer progression. *In vitro* experiments indicated that LAPTM4A may influence metastasis through the EMT pathway in glioma. Additionally, we found that LAPTM4A was associated with the tumor microenvironment (TME) and immunotherapy. Notably, drug sensitivity analysis revealed that patients with high LAPTM4A expression were sensitive to doxorubicin, which contributed to a reduction in LAPTM4A expression. Finally, we uncovered the FGD5-AS1-hsa-miR-103a-3p-LAPTM4A axis as a facilitator of glioma progression.

Conclusions: In conclusion, our study identifies LATPM4A as a promising biomarker for prognosis and immune characteristics in glioma.

## INTRODUCTION

Gliomas, malignant brain tumors, are increasingly prevalent among adults and characterized by their aggressive growth [1]. Due to the aggressive nature of gliomas, complete removal of glioma tissue is impractical [2, 3]. Furthermore, radiation resistance may result from continued treatment. Gliomas used to be categorized as either high-grade gliomas (HGG) or low-grade gliomas (LGG) according to the degree of aggressiveness. Pathology grading typically ranges from I to IV, with grade IV indicating glioblastoma (GBM), a type of glioma. Glioblastoma (GBM) has a 5-year survival rate of just 6.8%, making it one of the most severe malignant solid tumors [4]. Due to the varying levels of infiltration and individualized heterogeneity of glioma patients, even with the latest treatment modalities, including surgery, radiotherapy, and temozolomide (TMZ) chemotherapy, patients still have a high rate of recurrence [5]. Previous studies have identified several molecular markers, such as mutations and deletions of isocitrate dehydrogenase (IDH), for the pathological diagnosis and prognostic assessment of glioma patients [6]. However, obtaining information on the IDH genotype requires invasive procedures, such as surgery or biopsy, followed by laboratory tests, which are time-consuming [7]. While emerging biomarker studies have partially enhanced the diagnosis and treatment strategies for glioma, the extensive proliferation, invasiveness, angiogenesis, immunosuppression, and resistance to conventional treatment make glioma difficult to treat and have yet to yield satisfactory results [8].

Immunotherapy is gaining popularity in the field of oncology. In recent years, immune checkpoint blockers (ICBs) have made significant advancements in the treatment of various malignant solid tumors [9]. Targeting tumor immune checkpoints, such as programmed cell death protein 1 ligand (PD-L1) and cytotoxic T lymphocyte antigen 4 (CTLA-4), holds great promise for oncology treatment, particularly in combating glioma immune evasion [10]. However, the effectiveness of combining anti-PDL1 and anti-CTLA-4 therapies remains limited [11]. Therefore, it is crucial to further explore the role of biomarkers in the tumor immune microenvironment.

The lysosomal-associated protein transmembrane (LAPTM) family, which encompasses LAPTM4A, LAPTM4B, and LAPTM5, has been implicated in cell proliferation and carcinogenesis. Overexpression of the LAPTM4 family has been observed in several tumor types, including liver, breast, and gastric cancers, suggesting their involvement in tumorigenic processes [12]. Notably, high LAPTM5 expression has been associated with improved survival in patients with CD40-positive glioblastomas [13]. LAPTM4A, a gene encoding a protein involved in the translocation of small molecules across endosomes and lysosomal membranes, has shown promise as a reliable predictor of outcomes in patients with pancreatic adenocarcinoma when combined with nine other genes [14]. However, the role of LAPTM4A in gliomas remains largely unknown, with limited biomedical research conducted on this topic. Therefore, our study aims to investigate the prognostic value of LAPTM4A in glioma patients and shed light on its biological functions within gliomas.

In our study, we aimed to uncover novel biomarkers for glioma by employing the WGCNA method and analyzing data from the TCGA and the GTEx databases. Through this approach, we successfully identified LAPTM4A as a gene of significant clinical importance. To further assess its prognostic value, we conducted an evaluation and discovered that LAPTM4A has strong potential in this regard. Moreover, our analysis revealed that LAPTM4A is primarily associated with immunity and is expected to become an encouraging therapeutic target for tumor immunotherapy.

## MATERIALS AND METHODS

### Data acquisition and processing

The RNAseq data from the TCGA-GBM and TCGA-LGG projects, processed using the STAR pipeline, along with clinical data, were downloaded and organized from the TCGA database (https://www.cancer.gov/ccg/research/genome-sequencing/tcga) [15] using R packages “stats (4.2.1)” and “car (3.1–0)”. The extracted data were in FPKM format. In total, there were 706 samples, consisting of 701 neuroglioma patients and 5 normal tissue samples. Among the neuroglioma patients, there were 532 samples classified as low-grade glioma and 174 samples as glioblastoma multiforme. Since the TCGA database only contained 5 normal samples, we supplemented the analysis by including an additional 1152 normal brain tissue samples from the GTEx database (https://www.gtexportal.org/) [16]. The CGGA mRNAseq_693 data set, obtained from the CGGA database (http://www.cgga.org.cn/), was used as a validation cohort. Additionally, in order to obtain the LAPTM4A expression of distinct subtypes, we downloaded and analyzed the Bao, Phillips, and Rembrandt data set of the Gliovis website (http://gliovis.bioinfo.cnio.es/) [17].

### Weighted gene co-expression network analysis (WGCNA)

Weighted Gene Co-expression Network Analysis (WGCNA) was used to identify clusters (modules) of highly correlated genes, establish module-to-module relationships (using the feature gene network approach), and calculate module membership metrics for the identification of candidate biomarkers or therapeutic targets [18]. First, sample clustering of all DEmRNAs was applied to ensure the inclusion of high-quality samples with reliable RNA. Subsequently, a soft-thresholding power value of 12 (mRNA) (scale-free R^2^ = 0.8) was chosen to determine the scale-free topology model. The adjacency matrix was transformed into a topological overlap matrix (TOM). Based on the dissimilarity measurements from TOM, the mRNAs were grouped into different modules. A minimum module size of 30 and a cut height of 0.45 were applied to define the key modules. Module Eigengenes (MEs) were considered the major principal components of a given RNA module, while module membership (MM) was used to assess their correlation with gliomas. Furthermore, the turquoise module was determined as the most significant module correlation. Pearson algorithm was employed to calculate the connectivity of genes and genes with high connectivity were identified as hub genes for the module.

### DNA methylation and genetic alterations of LAPTM4A

MethSurv (https://biit.cs.ut.ee/methsurv/) [19] can combine DNA methylation data for multivariate survival analysis. To evaluate the prognostic values (OS) of CpG methylation of LAPTM4A in LGG and GBM, we entered “LAPTM4A” in the “Query” module, and selected an accessible CpG methylation site to analyze. Moreover, the methylation heatmap of LAPTM4A can be found in the “Gene visualization” module.

What’s more, the genomic profiles of LAPTM4A in cBioPortal (https://www.cbioportal.org) [20] were analyzed utilizing three datasets: Brain Lower Grade Glioma (TCGA, Firehose Legacy), Glioblastoma Multiforme (TCGA, Firehose Legacy), and Merged Cohort of LGG and GBM (TCGA, Cell 2016). We entered “LAPTM4A” in the “Query” module, and the LAPTM4A loci, type, and number of variants can be found in the “Cancer Type Summary” and “Mutation” modules.

### Protein interaction, structure and docking analysis

In the present study, GeneMANIA (http://genemania.org/) [21] was initially utilized to construct an interactive functional network for LAPTM4A.

Through the utilization of the cBioPortal portal website (https://www.cbioportal.org/), we investigated the secondary structures of LAPTM4A, MCOLN1, and IGF2BP3 by leveraging samples from a merged queue consisting of LGG and GBM entities (study ID, Merged Cohort of LGG and GBM).

Moreover, we employed the Protein Data Bank (PDB; https://www.rcsb.org/) to identify the advanced structures of MCOLN1 and IGF2BP3 (with PDB IDs: 5TJA and 6FQ1, respectively). Furthermore, the AlphaFold Protein Structure Database (https://swissmodel.expasy.org/) [22] was used to predict the advanced structure of LAPTM4A (ID: AF-Q15012-F1).

Finally, HDOCK (http://hdock.phys.hust.edu.cn/) [23] was employed to predict the interaction docking patterns among LAPTM4A, MCOLN1, and IGF2BP3 and visualized them using PyMOL software.

### Functional relevance analysis

CancerSea is a database for examining the biological role of target genes in tumor cells in the single-cell pattern (http://biocc.hrbmu.edu.cn/CancerSEA/) [24]. We entered “LAPTM4A” in the “Query” module. Subsequently, the single-cell sequence data in the Correlation Graph module were analyzed for correlations between LATPM4A and 14 cancer functional states. LATPM4A and function were screened for correlations at a *p*-value of 0.05.

LinkedOmics is a general online website covering 32 TCGA carcinoma-related datasets (http://www.linkedomics.org/login.php) [25]. The “HiSeq RNA” platform and the “TCGA_ GBMLGG” cohort were selected for analysis. The correlation between LAPTM4A and co-expressed genes was detected by Spearman’s test. Our team utilized the LinkFinder module in Variomics to study differentially expressed genes associated with LAPTM4A in TCGA GBMLGG.

### Single-cell sequencing analysis and immunoassay

TISCH (http://tisch.comp-genomics.org/home/) is a collection of scRNA-seq data for multiple cancer types that allows for cell type-specific analysis of target gene expression based on single-cell TME expression [26]. The following were our analysis parameters: LAPTM4A, major lineage, and all cancers. The heatmaps, scatter plots, and violin plots quantified and visualized the expression levels of LAPTM4A in each cell type. The TIMER2 database (http://timer.cistrome.org/) [27] studies immune infiltration of multiple cancer types systematically. We investigated the relationships between the expression of LAPTM4A and 12 immune cell subgroups, including cancer-associated fibroblasts (CAF), regulatory T cells (Tregs), macrophages, monocytes, and so on. Additionally, TISIDB, an integrated knowledge base portal (http://cis.hku.hk/TISIDB/) [28], is crucial in identifying how cancer and the immune system interact. To investigate the association between LAPTM4A and the expression of MHC, chemokines, chemokine receptors, immunostimulators, and immune inhibitors, we evaluated the expression levels of chemokines/chemokine receptors in TIIC by the corresponding module. Furthermore, we utilized the Wilcoxon test to investigate the correlation between LAPTM4A expression and immune checkpoint genes to guide clinical immunotherapy. The Tumor Immune Dysfunction and Exclusion (TIDE) algorithm was utilized to forecast possible immune checkpoint inhibitor (ICI) reactions.

### Drug sensitivity analysis

First of all, we utilized the Comparative Toxicogenomics Database (CTD, http://ctdbase.org/) [29] to query chemical agents that affected the expression of LAPTM4A. Subsequently, GSCALite (http://bioinfo.life.hust.edu.cn/web/GSCALite/) [30], a general site for the study of immune infiltration and drug sensitivity was applied to seek the drugs relevant to LAPTM4A. Additionally, we predicted chemotherapy response based on the Genomics of Drug Sensitivity in Cancer (GDSC, https://www.cancerrxgene.org/) [31, 32] and conducted the procedure through the R package ‘pRRophetic’. Ridge regression was employed to estimate the half-inhibitory concentration. Eventually, we calculated the association between LAPTM4A expression and the IC_50_ of anticancer drugs.

### Prediction and construction of ceRNA networks

The TargetScanHuman 8.0 (http://www.targetscan.org) [33], DIANA-microT (http://diana.imis.athena-innovation.gr/DianaTools/index) [34], and RNAinter (http://www.rnainter.org) [35] online websites are employed to forecast and analyze the miRNA potentially bound to the LAPTM4A. Next, we use miRNet 2.0 (https://www.mirnet.ca/miRNet/home) [36] and starBase 3.0 (https://rnasysu.com/encori/) [37] for prediction and analysis of the target lncRNA of the relevant miRNA to establish a possible ceRNA network.

### Cell culture

The U251 glioblastoma cell line was obtained from the Chinese Academy of Sciences in Shanghai, China. U251 cells were cultivated in RPMI-1640 medium, while the remaining cell lines were cultured in DMEM supplemented with 10% fetal bovine serum and 1% penicillin-streptomycin. All cell cultures were maintained at 37°C in a CO_2_ incubator with 5% carbon dioxide.

### The small interfering RNA (siRNA) and plasmid transfection

The siRNA was purchased from Hanbio (Shanghai, China) and transfected with liposomes 3000 (Shanghai, China). Cells were cultured in a 6-well plate until they reached a density of 50–60% during the logarithmic growth phase. 5 μL of liposomes 3000 and 125 μL Opti-MEM solutions were mixed. DNA (2 μg) and P3000 reagent (10 μl) were diluted to a final volume of 125 μl using Opti-MEM solution. The DNA-lipid complex was incubated at room temperature for 10–15 minutes before being added to the cells. The cells were then incubated at 37°C for 2–4 days [38].

### Real-time PCR (qRT-PCR)

Total RNA extraction was carried out using the Trizolrogen (USA) protocol. The extracted RNA was subsequently converted into complementary DNA (cDNA) using an RT-PCR kit. To assess the mRNA levels of the target genes, a Real-Time qPCR kit was employed.

### Western blotting

The RIPA protein extraction method (Beyotime, Shanghai, China) was used to extract total protein from glioblastoma cells. Following centrifugation, the protein concentration was determined with the BCA protein assay kit (Thermo Fisher Scientific, Waltham, MA, USA). For protein separation, SDS-polyacrylamide gel electrophoresis (SDS-PAGE) was employed, and PVDF membrane was utilized for protein transfer. After incubating the membrane overnight at 4°C with the specified antibodies, it was washed three times with TBST. Subsequently, the membrane was incubated with LAPTM4A-conjugated secondary antibodies at room temperature for the next hour. Finally, protein expression was detected using the Amersham™ Image Quant 800 system (GE Healthcare, Chicago, IL, USA).

### Transwell assay

To evaluate cell invasion, Transwell chambers (Shanghai, China) were utilized. In a nutshell, cells were seeded in the upper chamber of Transwell coated with a matrix gel in serum-free DMEM medium at a concentration of 1 mg/ml. Once cell confluence reached around 80%, DMEM medium supplemented with 10% fetal bovine serum (FBS) was added to the lower chamber, and cells were cultured for 24 hours. Following this, invasive cells were fixed with 4% paraformaldehyde and stained with 0.5% crystal violet for 30 minutes. The invaded cell count was assessed using an Olympus microscope to determine the cell’s invasion capability.

### Dual-luciferase reporter gene system

The sections encompassing the binding sites of miR-103a-3p or the respective mutants from FGD5-AS1 and LAPTM4A were cloned into a pmiRGLO Vector (Shanghai, China). The vectors mentioned above, together with the miR-103a-3p mimic and inhibitor, were co-transfected into U251 cells using Lipofectamine 2000 (Shanghai, China). The dual-luciferase reporter gene system (Shanghai, China) was used to assess luciferase activities.

### Statistical analysis

We utilized R (version 4.1.2) for the ensuing analysis. We extracted the expression data of LAPTM4A from the normal and tumor tissues in LGG, GBM, and GBMLGG of TCGA and GTEx. The RNAseq data and clinical data were downloaded in level 3 HTSeq-FPKM format in the TCGA GBMLGG project, and three data points, WHO grade, IDH mutation status, and 1p/19q deletion, were obtained from the study by Ceccarelli et al. The visualization was performed using the ggplot2 package. We utilized the R package “survival” to construct COX proportional hazards regression models investigating the relationship between LAPTM4A expression and prognosis in glioma and performed statistical tests using the log-rank test to obtain prognostic significance [39]. *P*-values < 0.05 were considered significant (^*^P < 0.05, ^**^P < 0.01, ^***^P < 0.001).

### Availability of data and materials

The datasets used and/or analyzed during the current study are available from the corresponding author upon reasonable request.

## RESULTS

### Constructing WGCNA and identifying LAPTM4A

**Figure 1:**
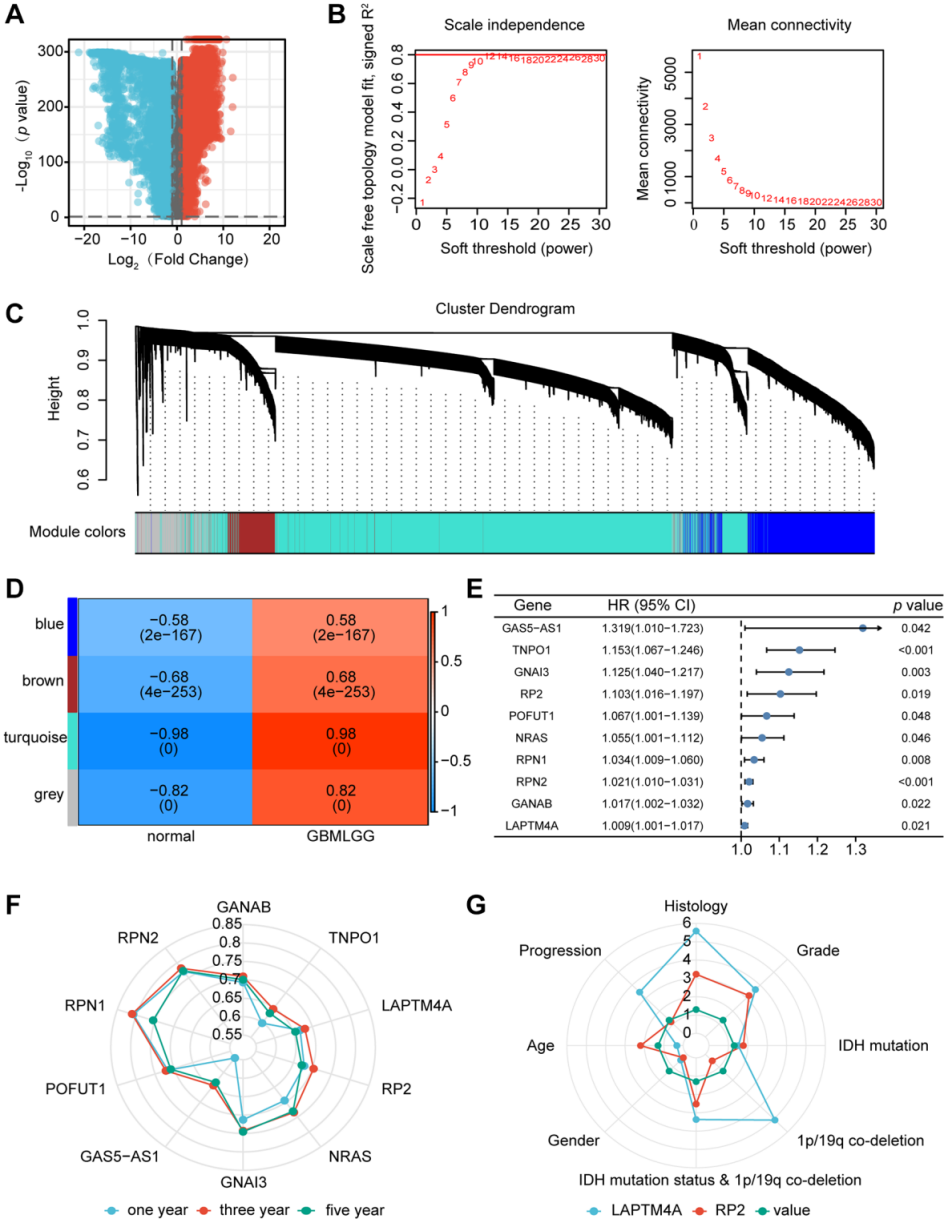
**Identification of the key gene modules in WGCNA.** (**A**) The volcano map showed differentially expressed genes. (**B**) Determination of the soft-thresholding power. (**C**) Dendrogram of differentially expressed genes clustered based on a dissimilarity measure (1-TOM). (**D**) The correlation of gene modules with clinical traits. (**E**) Gene correlation scatter plot of the turquoise module. (**F**) The 1-, 3-, and 5-years ROC for the top ten genes. (**G**) Comparison of the clinical significance of LAPTM4A and RP2.

Initially, we extracted a total of 30,029 genes from the TCGA-GBM, TCGA-LGG, and GTEx-brain datasets for our analysis, as depicted in Figure 1A. Through rigorous screening, we identified 8,762 genes (meeting the criteria of positive logFC and *p*-value < 0.05) for subsequent WGCNA analysis. To establish a scale-free topological network, we applied a soft threshold power (β) of 12, as shown in Figure 1B. The resulting hierarchical clustering tree revealed four distinct co-expression modules, as depicted in Figure 1C. Notably, the turquoise module demonstrated the strongest positive correlation (R = 0.98, *p* < 0.001) with the tumor proportion, as illustrated in Figure 1D. This module encompassed a total of 5,679 genes (Supplementary Table 1).

We further selected the top 5% hub genes (Supplementary Table 2) and identified 50 genes associated with glioma patient survival using HR >1 and *p* < 0.05 as criteria. Through univariate and multivariate COX regression analysis, as well as ROC survival curves, we determined that 12 out of the 50 genes exhibited superior predictive capability for 1-year and 5-year survival in glioma patients, as presented in Figure 1E. Notably, of these rigorously selected genes, RP2 and LAPTM4A demonstrated not only independent prognostic values for glioma patients but also displayed high Area Under ROC Curve (AUC) values. Importantly, these two genes have not been previously reported or studied extensively in the context of glioma (refer to Figure 1F).

Furthermore, we investigated the correlation between the expression of RP2 and LAPTM4A and the clinicopathological features of glioma patients using the CGGA database. Interestingly, we observed that LAPTM4A exhibited greater clinical significance (Figure 1G). Ultimately, LAPTM4A was identified as the gene of primary interest in our study.

### Expression and clinical parameters of LAPTM4A in glioma

**Figure 2:**
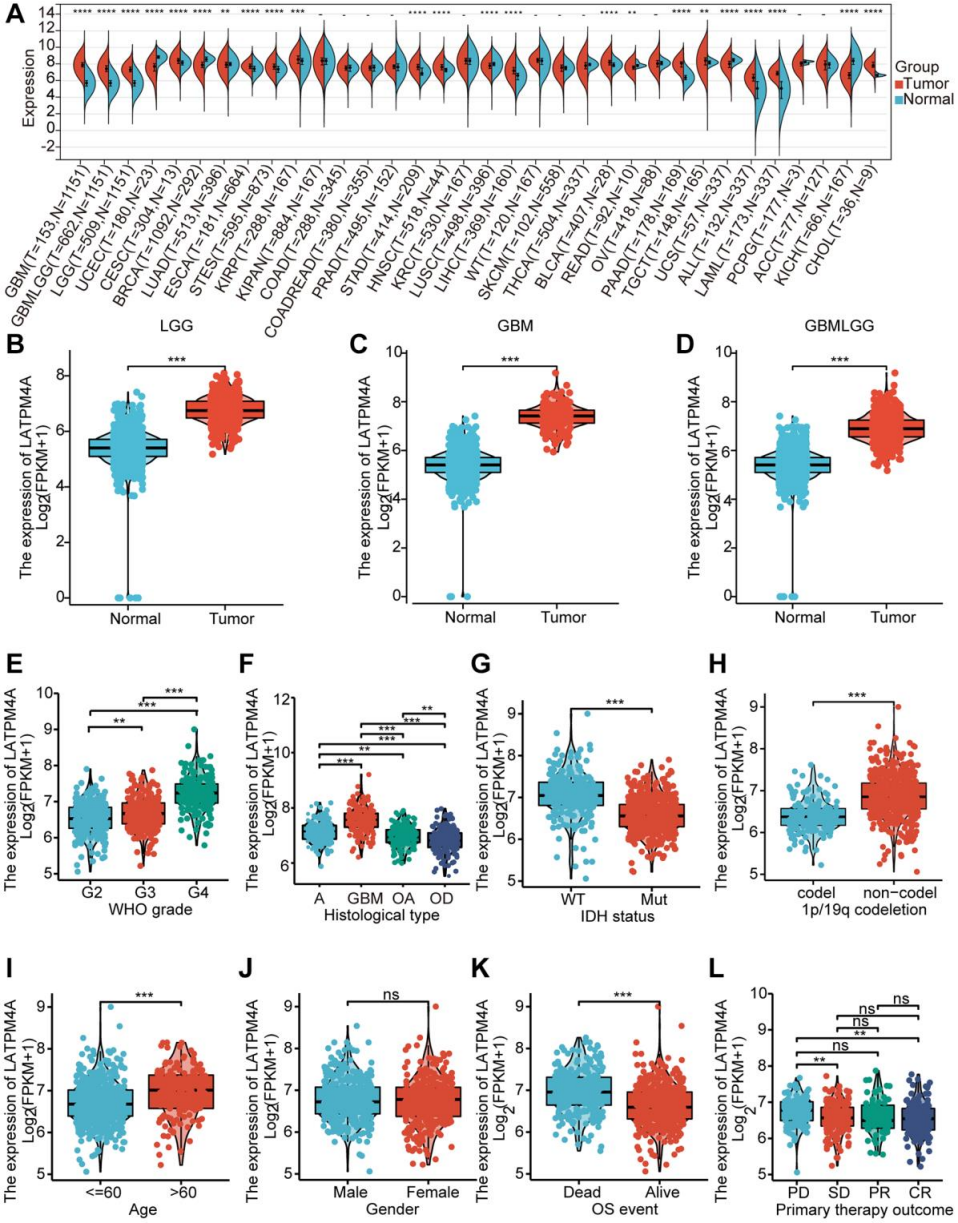
**Expression of LAPTM4A in glioma.** (**A**) The expression level of LAPTM4A in different types of tumor tissues and normal tissues in the TIMER database. (*p* < 0.05) (**B**–**D**) Expression levels of LAPTM4A were higher than corresponding normal tissues in LGG, GBM, and GBMLGG samples. The box plot showed the association of LAPTM4A expression with clinicopathological characteristics. (**E**) WHO grade, (**F**) Histological type, (**G**) IDH status, (**H**) 1p/19q codeletion, (**I**) Age, (**J**) Gender, (**K**) OS event, (**L**) Primary therapy outcome.

To determine the expression level of LAPTM4A, we conducted a comprehensive analysis of LAPTM4A mRNA levels using data from the TCGA and GTEx databases. Our results, as depicted in Figure 2A, demonstrated a significant increase in LAPTM4A expression in 16 different types of carcinomas, with particularly high expression observed in LGG, GBM, and GBMLGG. This upregulation of LAPTM4A in LGG, GBM, and GBMLGG was further confirmed by box diagrams displayed in Figure 2B–2D. Moreover, in order to establish the association between LAPTM4A expression in GBMLGG and various clinic parameters, we assessed LAPTM4A expression levels in different cohorts stratified by WHO grade, histological type, IDH status, 1p19q codeletion, age, gender, OS event, and primary therapy outcome, utilizing the TCGA and GTEx databases (Figure 2E–2L). Our analysis revealed a significant correlation between LAPTM4A expression and clinicopathological features, with the exception of gender (*p* < 0.01). Notably, LAPTM4A expression exhibited an increasing trend as the WHO grade advanced. Furthermore, LAPTM4A was found to be overexpressed in IDH wild-type cases when compared to IDH mutant cases, and higher expression levels were observed in 1p19q non-co-deletion as opposed to co-deletion. Collectively, these findings suggest that LAPTM4A expression is elevated in gliomas and holds promise as a potential biomarker for assessing glioma progression.

### Over-expression of LAPTM4A predicted an unfavorable prognosis in glioma

**Figure 3:**
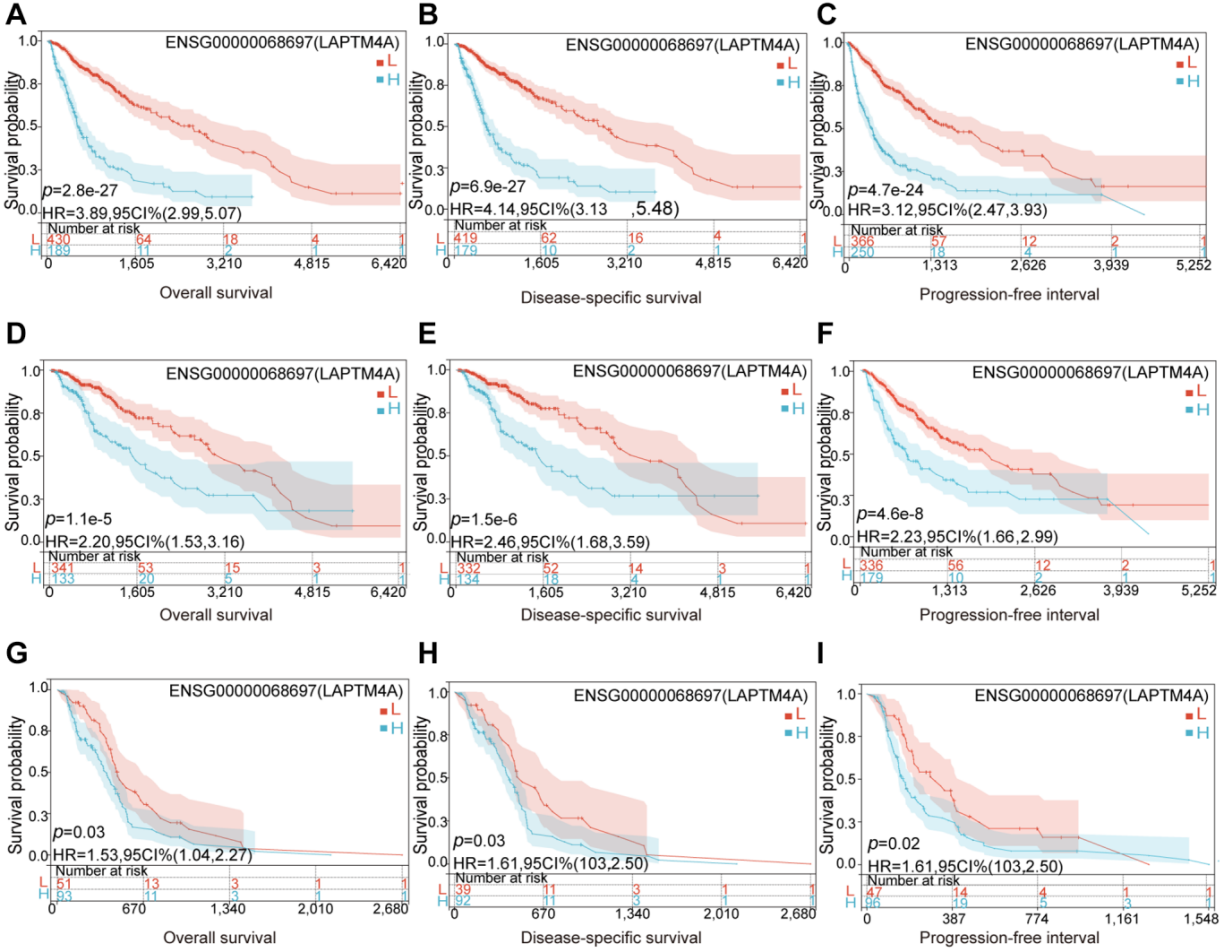
**Relationship between LAPTM4A and prognosis of glioma patients.** LGG patients with higher expression levels of LAPTM4A had unfavorable (**A**) OS, (**B**) DSS, and (**C**) PFS. GBMLGG patients with higher expression levels of LAPTM4A had awful (**D**) OS, (**E**) DSS, and (**F**) PFS. GBM patients with higher expression levels of LAPTM4A had undesirable (**G**) OS, (**H**) DSS, and (**I**) PFS.

Considering its significant expression in LGG, GBM, and GBMLGG, the prognostic and diagnostic value of LAPTM4A was assessed. Utilizing the TCGA and GTEx databases, we calculated the correlation between LAPTM4A expression in LGG, GBM, and GBMLGG, and patient prognosis, including overall survival (OS), disease-specific survival (DSS), and progression-free interval (PFI). Our findings revealed a significant association between overexpression of LAPTM4A and poor OS (*p* = 2.8 e-27) and DSS (*p* = 6.9 e-27) as well as PFI (*p* = 4.7 e-24) in GBMLGG (Figure 3A–3C). Similarly, overexpression of LAPTM4A was associated with unfavorable OS (*p* = 1.1 e-6) and DSS (*p* = 1.5 e-6), and PFI (*p* = 4.8 e-6) in LGG (Figure 3D–3F), and correlated with poor OS (*p* = 0.03) and DSS (*p* = 0.03) and PFI (*p* = 0.02) in GBM (Figure 3G–3I). The validation of survival across all WHO grades of primary (*p* = 0.028) and recurrent glioma (*p* = 0.04) was performed using the CGGA database (Supplementary Figure 1A), yielding consistent results. Furthermore, the area under the ROC curve was 0.982, 0.992, and 0.984 in LGG, GBM, and GBMLGG, respectively, indicating superior diagnostic accuracy for LAPTM4A (Supplementary Figure 1B–1D). Subsequently, we developed a nomogram integrating the significant clinic parameters mentioned above to estimate the survivability of GBMLGG patients (Supplementary Figure 1E). The predictive performance of the nomogram was evaluated using calibration curves, which demonstrated close agreement between predicted and actual 1, 3, and 5-year survival durations (Supplementary Figure 1F). In conclusion, our findings support the conclusion that overexpression of LAPTM4A is predictive of an unfavorable prognosis in gliomas.

### Association of LAPTM4A with DNA methylation and genetic alterations in glioma

To elucidate the underlying cause of LAPTM4A dysregulation, we conducted investigations into DNA methylation and genetic variation of LAPTM4A. Our study aimed to determine the methylation status of LAPTM4A across different subtypes and WHO grades of glioma. Boxplots were employed to visualize the variations in LAPTM4A methylation levels among the subtypes and WHO grades (Supplementary Figure 2A, 2B). Intriguingly, high-grade gliomas exhibited lower levels of methylation compared to low-grade gliomas. Furthermore, we utilized the MethSurv network tool to delve deeper into the methylation patterns of LAPTM4A in GBM and LGG. The resulting heatmap illustrated a predominance of hypomethylation at most sites within LAPTM4A (Supplementary Figure 2C, 2D). Notably, hypomethylated sites, such as cg11645081, were associated with poor survival outcomes in LGG (Supplementary Figure 2E). However, this phenomenon was more prevalent in GBM, as exemplified by sites cg04515480, cg10383839, and cg17989428 (Supplementary Figure 2F–2H). In conjunction with previous findings, we observed an increase in LAPTM4A expression with increasing grade, while the level of LAPTM4A methylation decreased with glioma grade.

Moreover, genetic variation plays a crucial role in gene expression and tumorigenesis. Through analysis using the cBioPortal website, we determined that the mutation rate of LAPTM4A in gliomas was generally low, with amplification being the most common alteration (Supplementary Figure 3A). However, a detailed examination of copy number variation revealed significant differences in LAPTM4A expression among neutral, gain, and loss groups in glioma (Supplementary Figure 3B). Notably, we discovered an association between LAPTM4A expression and neoplasm-related mutant genes in glioma. In GBM, the LAPTM4A high expression group exhibited a higher frequency of SSPO mutations and a lower frequency of TP53 and ATRX mutations (Supplementary Figure 3C). Similarly, in LGG, the LAPTM4A high expression group displayed a higher frequency of TP53, ATRX, and EGFR mutations, while having a lower frequency of IDH1, CIC, FUBP1, NOTCH1, and ZBTB20 mutations (Supplementary Figure 3D). Furthermore, in GBMLGG, the LAPTM4A high expression group demonstrated higher frequencies of TTN, PTEN, and EGFR mutations, and lower frequencies of IDH1, CIC, and FUBP1 mutations (Supplementary Figure 3E).

### LAPTM4A is associated with multiple immune-related and cancer-relevant pathways in glioma

**Figure 4:**
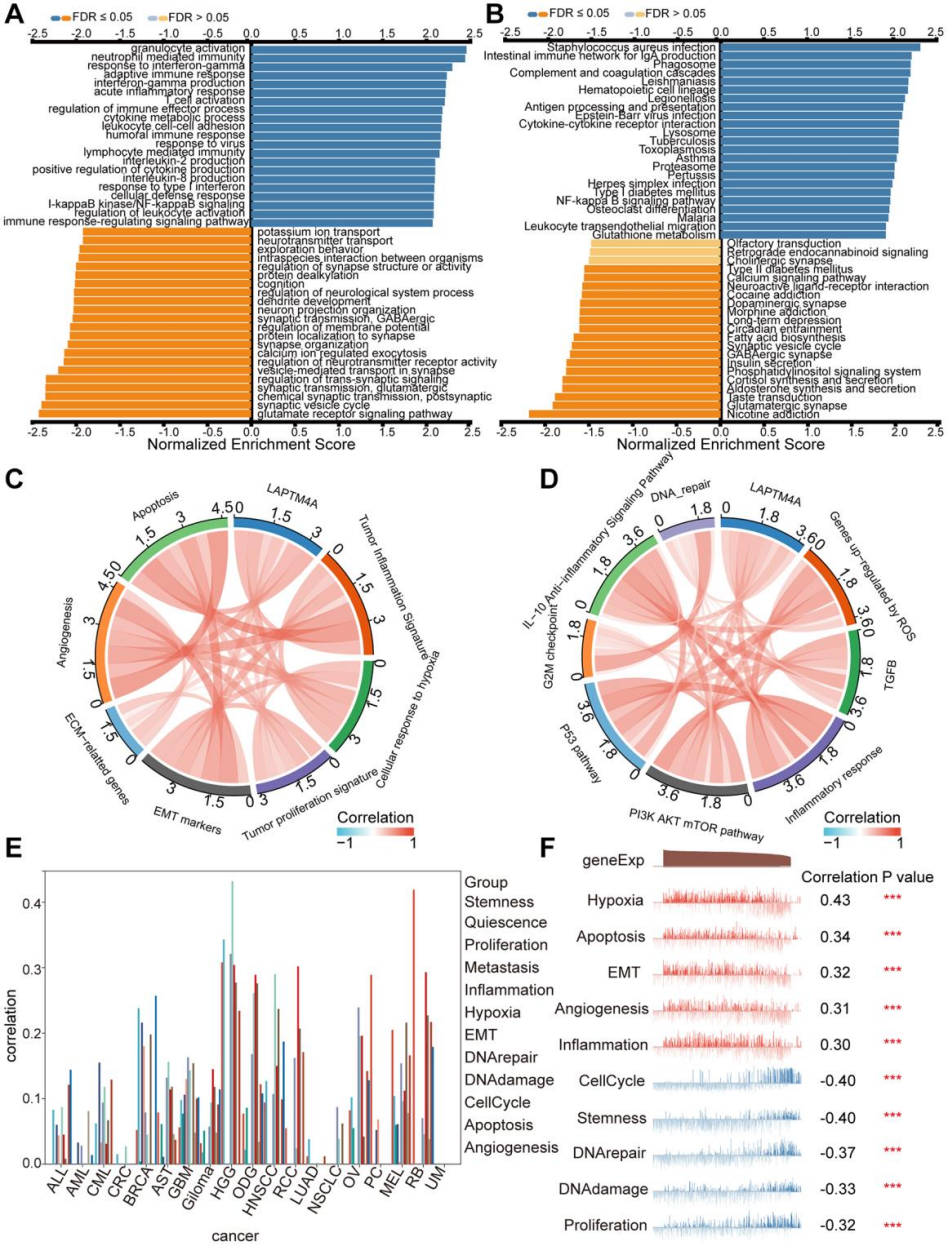
**Pathway enrichment analysis of LAPTM4A.** (**A**, **B**) Significantly enriched GO and KEGG pathways of LAPTM4A. GO: Gene Ontology; KEGG: Kyoto Encyclopedia of Genes and Genomes. (**C**, **D**) Correlation of LAPTM4A expression and cancer-related pathways. (**E**) Functional relevance of LAPTM4A in pan-cancers from cancerSEA. (**F**) Functional relevance of LAPTM4A in GBMLGG from cancerSEA red plots suggested a positive correlation, while blue plots suggested a negative correlation.

To gain further insights into the functional significance of LAPTM4A, a series of comprehensive analyses were conducted. Leveraging the LinkedOmics web resource, we performed GO pathway enrichment analysis, which revealed significant enrichment of LAPTM4A in various terms, including neutrophil-mediated immunity, acute inflammatory response, interferon-gamma production, control of humoral immune response, and immunological effector processes (Figure 4A). Additionally, KEGG pathway enrichment analysis highlighted the enrichment of LAPTM4A in specific pathways such as complement and coagulation cascades, the phagosome, antigen processing and presentation, cytokine-cytokine receptor interaction, and the lysosome (Figure 4B). Moreover, utilizing the TCGA database, we demonstrated the association between immune and cancer-associated pathways and LAPTM4A expression. Notably, LAPTM4A expression showed positive associations with cellular responses to hypoxia, apoptosis, the inflammatory response, angiogenesis, and epithelial-mesenchymal transition (EMT) (Figure 4C, 4D). To validate these findings, we further employed the CancerSea database to explore the comprehensive enrichment pathways in various glioma subtypes, which corroborated our previous results (Figure 4E). Notably, high-grade glioma (HGG) exhibited the strongest association with the aforementioned pathways (Figure 4F). In conclusion, LAPTM4A plays a significant role in multiple immune-related and cancer-relevant pathways in glioma.

### LAPTM4A may affect the invasion and migration through the EMT pathway in glioma

**Figure 5:**
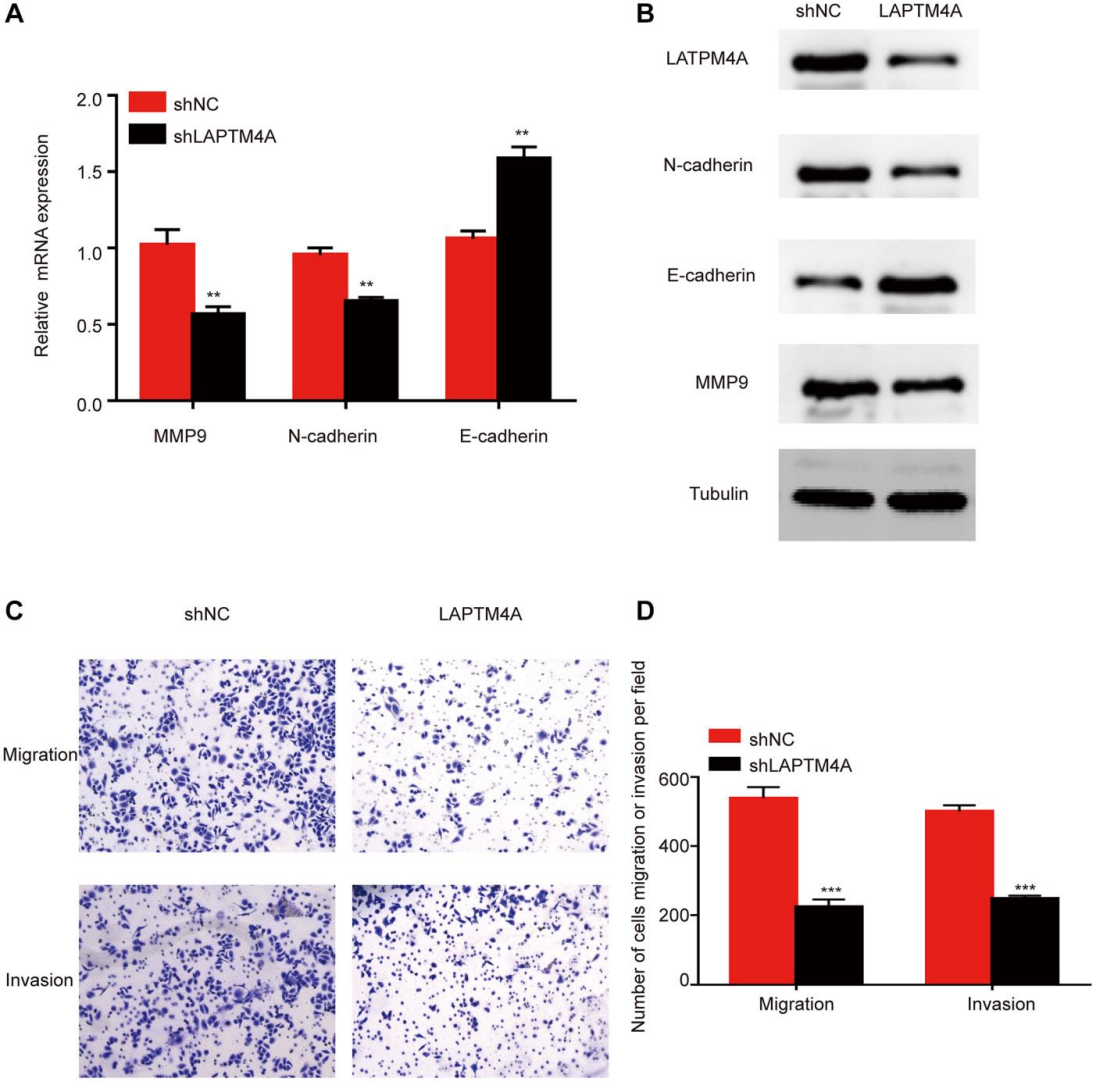
**LAPTM4A may affect the invasion and migration through the EMT pathway in glioma.** Changes in the expression of n-cadherin, e-cadherin, and MMP 9 after LAPTM4A knockdown (**A**) with mRNA aspect, (**B**) with protein aspect. (**C**, **D**) Effect of LAPTM4A knockdown on glioma cell invasion and migration.

Previously, the Cancer Genome Atlas Network classified glioblastoma multiforme into four distinct molecular subtypes: proneural, neural, mesenchymal, and classical [40]. Mesenchymal GBM cells are known to exhibit enhanced motility and invasion, in addition to higher expression levels of proteins related to cell movement, as compared to epithelial tumor cells. To explore the expression discrepancies of LAPTM4A among these subtypes, we assessed LAPTM4A expression levels in the Bao, Phillips, and glioma Rembrandt datasets. Our findings showed that mesenchymal subtype gliomas express LAPTM4A more frequently than other subtypes (Supplementary Figure 4A–4C). We further conducted a ROC analysis to evaluate the diagnostic power of LAPTM4A on the mesenchymal phenotype, which yielded an AUC of 0.859, 0.815, and 0.790 for LAPTM4A in Bao [41], Phillips [42], and Rembrandt [43], respectively, indicating its strong diagnostic potential (Supplementary Figure 4D–4F). To corroborate these results, we performed plasmid-mediated knockdown of LAPTM4A in the U251 cell line and assessed the expression of key molecules involved in epithelial-mesenchymal transition (EMT), such as N-cadherin, E-cadherin, and MMP9, using qRT-PCR and Western blotting. Interestingly, LAPTM4A knockdown resulted in higher levels of E-cadherin protein expression, while simultaneously lowering the protein levels of N-cadherin and MMP9 (Figure 5A, 5B). As EMT programming has been implicated in the metastasis of malignant tumor cells originating from epithelial cells [44], we further explored the relationship between LAPTM4A expression and tumor metastasis using the Transwell assay. The results demonstrated that the knockdown of LAPTM4A inhibited the invasion and migration of glioblastoma cells (Figure 5C, 5D). Collectively, our findings suggest that suppression of LAPTM4A expression may hinder glioblastoma invasion and migration through the EMT pathway.

### PPI network analysis and molecular docking model of LAPTM4A

Significant scientific advancements have shed light on the pivotal role of protein-protein interactions in various fundamental biological processes within living cells [45]. To explore this further, we utilized the GeneMANIA website to construct an interaction network involving LAPTM4A and other proteins (Supplementary Figure 5A). This analysis revealed that LAPTM4A physically interacts with six proteins. Of interest was the notable protein-protein interaction observed between LAPTM4A, MCOLN1, and IGF2BP3. Furthermore, we investigated the secondary structures of these proteins, including valuable information about chemical remodeling sites such as glycosylation, acetylation, ubiquitination, and phosphorylation, using the cBioPortal database (Supplementary Figure 5B). To gain insights into the three-dimensional structures of LAPTM4A, MCOLN1, and IGF2BP3, we employed the AlphaFold Protein Structure and PDB databases for tertiary structure prediction. Subsequently, employing the H-DOCK server, we predicted potential binding sites between LAPTM4A, IGF2BP3, and MCOLN1, as visually represented in Supplementary Figure 5C, 5D.

### Location and infiltration analysis of LAPTM4A in the tumor microenvironment integrating of single-cell sequencing analysis and the ESTIMATE algorithm

The intricate molecular compositions of both the internal and external tumor microenvironment, primarily comprised of immune and stromal cells alongside other associated cell types, play a crucial role in nurturing tumor growth. In order to identify the key cell types expressing LAPTM4A within the cancer microenvironment, we conducted a comprehensive single-cell analysis of LAPTM4A across 80 cancer sample datasets. By utilizing the TISCH online tool, we examined the expression levels of LAPTM4A in 34 distinct cell types, including immune cells, stromal cells, malignant cells, and functional cells, as depicted in the heatmap presented in Supplementary Figure 6A.

Notably, our findings highlighted a predominant expression of LAPTM4A in immune cells, particularly monocytes and macrophages, across various cancer types. This observation was consistent across multiple databases, such as GliomaGSE102130 (Supplementary Figure 6B) and Glioma_GSE131928_10X (Supplementary Figure 6C), where LAPTM4A was primarily expressed in monocytes/macrophages and AC-like malignant cell clusters.

**Figure 6:**
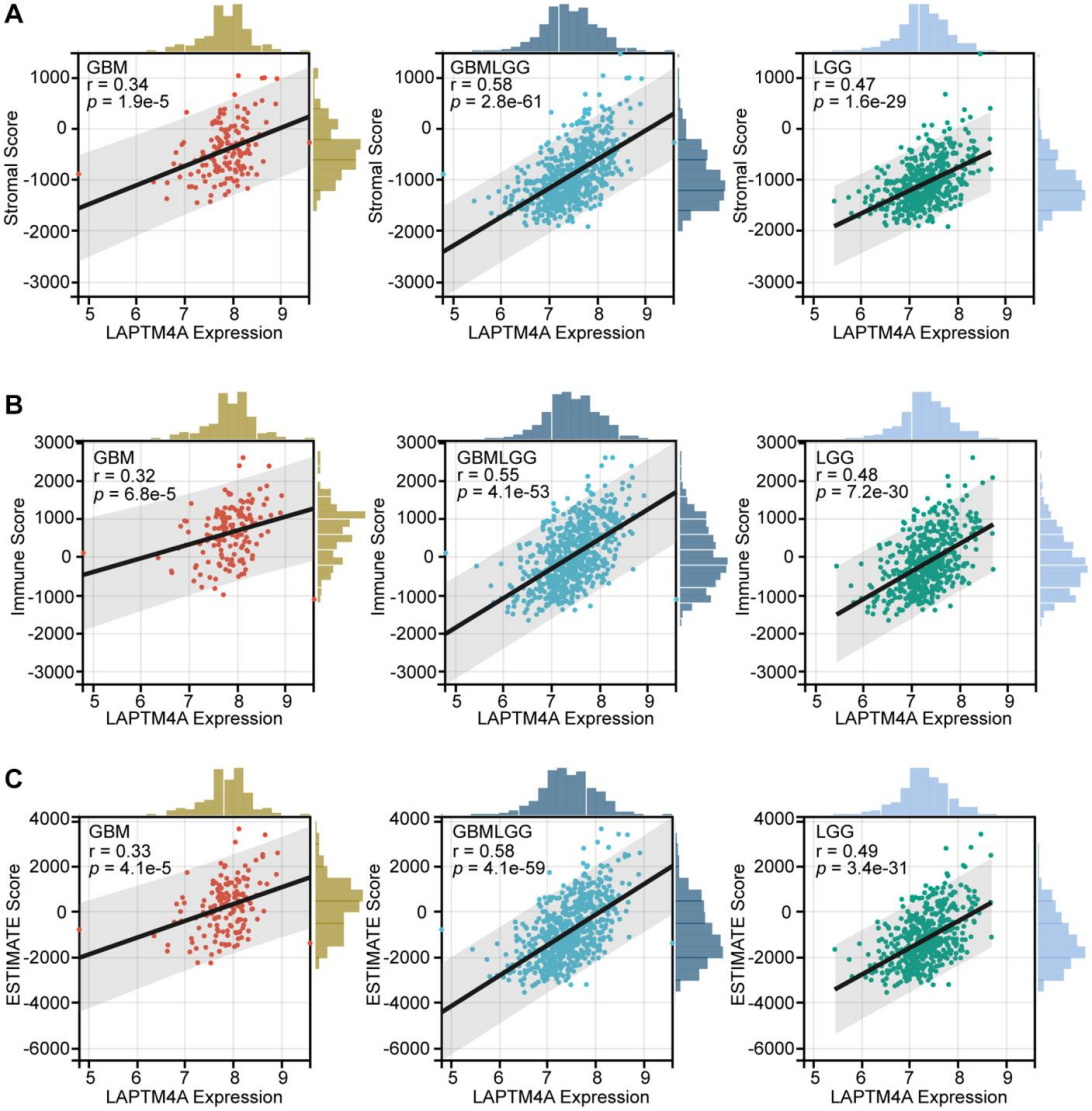
**The association between LAPTM4A with the tumor microenvironment.** (**A**) The relevance between LAPTM4A expression and the stromal score in GBM, LGG, and GBMLGG. (**B**) The relevance between LAPTM4A expression and the immune score in GBM, LGG, and GBMLGG. (**C**) The relevance between LAPTM4A expression and the ESTIMATE score in GBM, LGG, and GBMLGG.

Furthermore, to elucidate the relationship between LAPTM4A expression and the degree of infiltration within LGG, GBM, and GBMLGG, we performed correlation studies using stromal scores, immune scores, and ESTIMATE scores obtained from the ESTIMATE method (Figure 6). The results demonstrated a positive association between LAPTM4A expression and immunological, stromal, and estimation scores across LGG, GBM, and GBMLGG, with a particularly noteworthy correlation observed in GBMLGG (correlation coefficient >0.5). These findings further support the notion that LAPTM4A is predominantly expressed in monocytes/macrophages and AC-like malignant cells, and is intricately linked to the tumor microenvironment in gliomas.

### Relationship between LAPTM4A expression and the tumor immune cell infiltration and immune-related molecules

**Figure 7:**
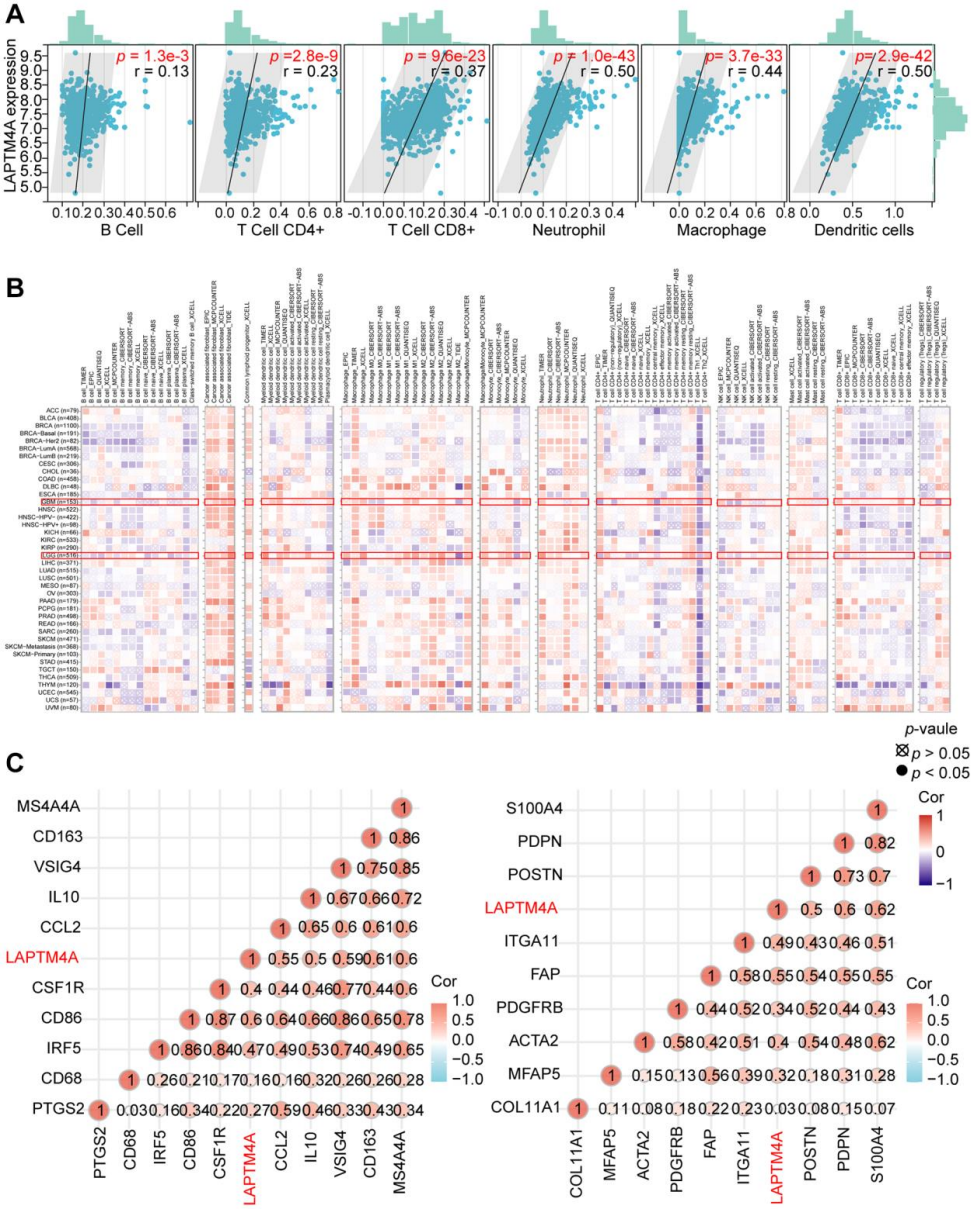
**Analysis of the correlation between LAPTM4A expression and immune cell infiltration.** (**A**) The relevance between LAPTM4A expression and the infiltration of five immune cells. (**B**) The association between LAPTM4A expression and the infiltration of various immune cells in pan-cancers. (**C**) The connection between LAPTM4A expression and several notable biomarkers of Macrophage/Monocyte and cancer-associated fibroblast.

Previous pathway enrichment analyses have established a strong association between LAPTM4A and immune-related pathways in gliomas. First, we utilized the TIMER method to investigate the relationship between LAPTM4A and immune infiltration in GBMLGG. Our results, depicted in scatter plots, revealed a significant association of LAPTM4A with neutrophils, macrophages, and dendritic cells in GBMLGG, and a mild correlation with B cells, CD4+ T cells, and CD8+ T cells (Figure 7A).

To further explore this relationship between LAPTM4A expression and immune cell subtypes in LGG and GBM, we utilized the TIMER2 website. Our results, as illustrated in the heatmap, demonstrated a positive correlation between LAPTM4A expression and the infiltration levels of common lymphoid progenitor, cancer-associated fibroblast, macrophage, monocyte, and neutrophil in LGG, and macrophage, common lymphoid progenitor, and cancer-associated fibroblast in GBM. Conversely, LAPTM4A was negatively correlated with NK cells and T cells CD4+ Th1 in LGG and GBM (Figure 7B).

Given the strongly positive association between LAPTM4A and monocytes/macrophages, as well as cancer-associated fibroblasts, we further examined the relationship between LAPTM4A and their biomarkers at the mRNA level (Figure 7C). To comprehensively investigate the link between LAPTM4A and immune system function, we performed gene co-expression analyses of LAPTM4A and immune-involved genes, including MHC, immunological activation, immunosuppression, chemokine, and chemokine receptor genes. Our outcomes revealed that the majority of immune-related molecules were co-expressed with LAPTM4A and positively correlated with LAPTM4A in gliomas (Supplementary Figure 7). Taken together, our findings suggest that LAPTM4A plays an essential role in immune infiltration and immune system function in gliomas.

### Association between LAPTM4A expression and immunotherapy

**Figure 8:**
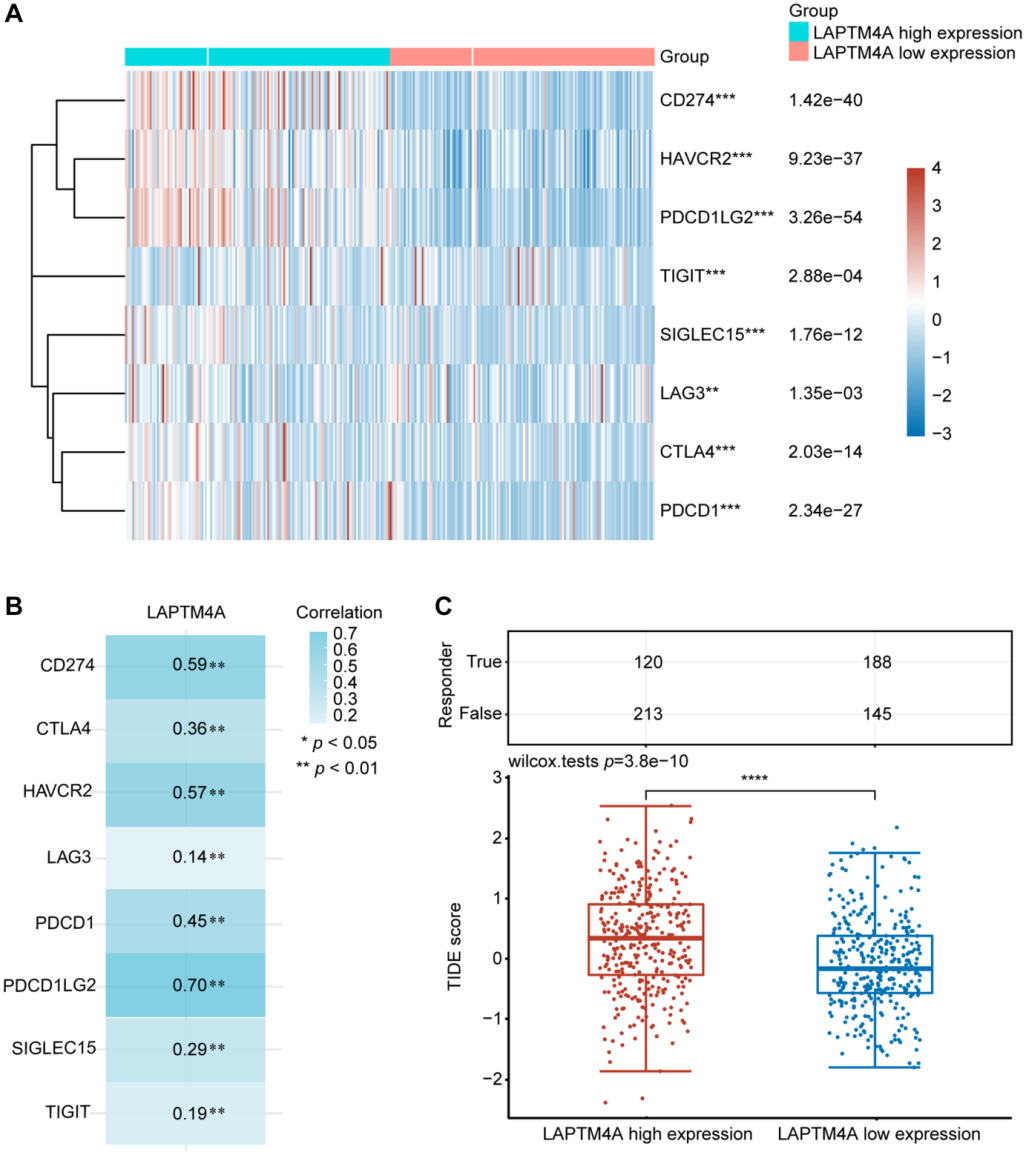
**The relationship between LAPTM4A expression and immunotherapy.** (**A**) LAPTM4A differential expression status of the immune checkpoint genes under the high and low expression groups. (**B**) The correlation between LAPTM4A and the immune checkpoint genes. (**C**) The TIDE score of the LAPTM4A.

Immunotherapy has emerged as a pivotal approach in the management of carcinoma. To delve into the association between LAPTM4A and immunotherapy, we focused on the expression patterns of eight immune checkpoint (ICP) genes in relation to LAPTM4A expression levels. Our findings demonstrated that when LAPTM4A was highly expressed, there was a corresponding upregulation of immune checkpoint genes (Figure 8A, 8B). Notably, LAPTM4A exhibited a strong correlation with PDCD1LG2, CD274, and HAVCR2.

To gain a deep understanding of the impact of LAPTM4A expression on the response to immune checkpoint blockade (ICB) treatment, we determined the TIDE scores for GBMLGG using the TIDE algorithm. It is worth noting that high TIDE scores are indicative of a diminished response to ICB therapy and shorter survival following such therapy. Figure 8C showed that the LAPTM4A high expression group exhibited higher TIDE scores in GBMLGG, thus classifying LAPTM4A as a risk factor (Figure 8C). In summary, the downregulation of LAPTM4A expression may hold promise for enhancing the efficacy of immunotherapy in glioma patients.

### Drug sensitivity analysis of LAPTM4A

**Figure 9:**
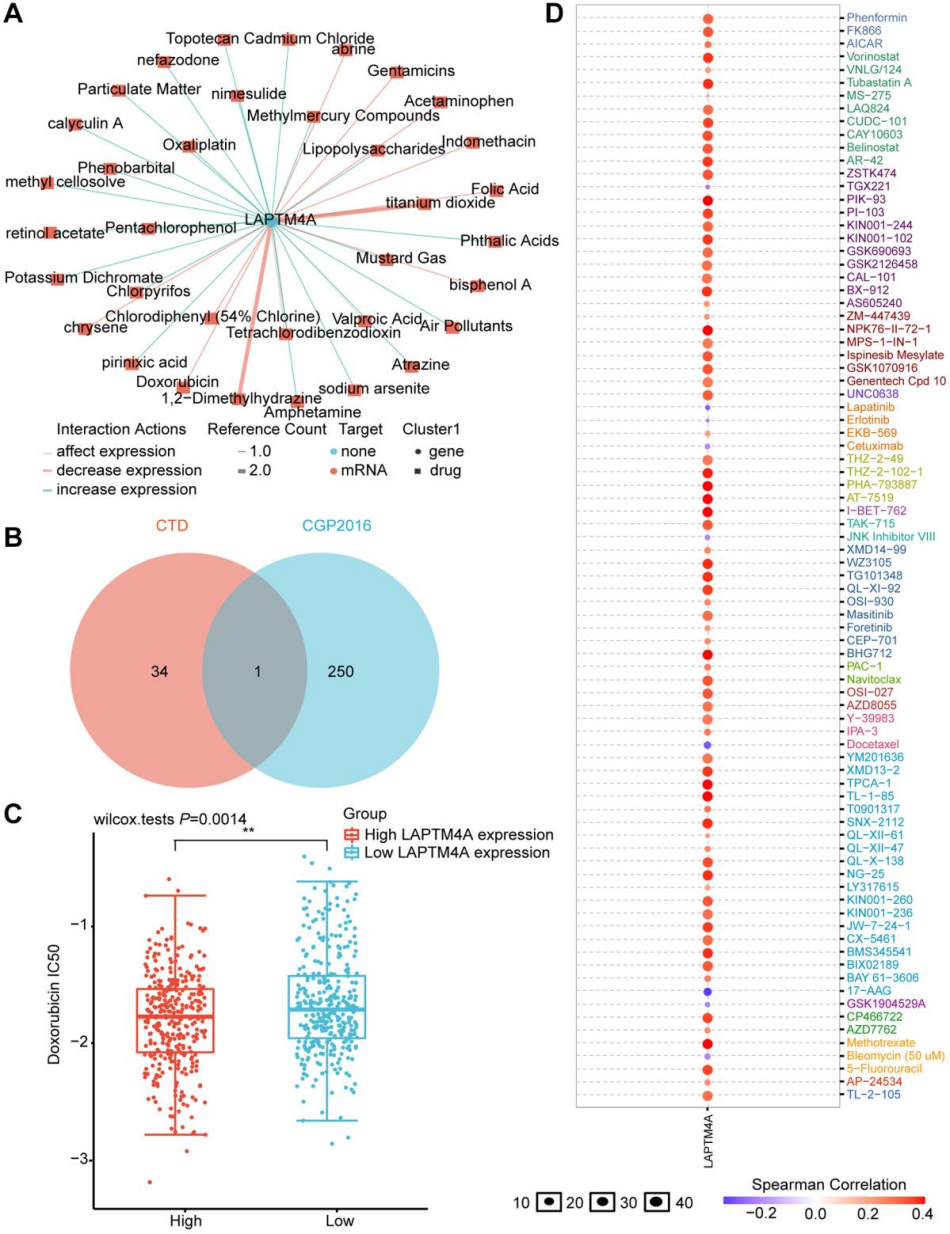
**Prediction of LAPTM4A expression-related drugs.** (**A**) An advanced network diagram shows 37 cancer-related drugs that can modulate LAPTM4A expression. (**B**) A Venn diagram demonstrates drugs related to LAPTM4A expression in CTD and cgp2016. (**C**) Relationship between LAPTM4A expression and IC50 of doxorubicin. (**D**) LAPTM4A is resistant to 73 drugs and sensitive to 9 drugs.

To evaluate the influence of LAPTM4A on drug resistance in patients, we conducted an in-depth analysis of the correlation between LAPTM4A expression and multidrug sensitivity using the CTD and GSCALite platforms. Our study revealed that the expression of LAPTM4A was under the regulation of 35 small-molecule drugs (Figure 9A). Leveraging the CGP2016 database, we further examined the IC50 values of drugs based on LAPTM4A expression. Venn diagrams created with CTD and CGP2016 data showed that a specific medication has the capacity to downregulate LAPTM4A expression (Figure 9B), making patients more receptive to doxorubicin in cases where LAPTM4A expression was high (Figure 9C).

We also harnessed the power of the GSCA website to investigate the drug sensitivity associated with LAPTM4A expression. Remarkably, patients with high levels of LAPTM4A expression were found to be resistant to 41 small-molecule drugs (Figure 9D). An additional Venn diagram, integrating data from GSCA and CGP2016, revealed a total of eight drugs, seven of which were consistent with our previous results and exhibited sensitivity in patients with high LAPTM4A expression (Supplementary Figure 8).

These findings not only enhance our understanding of the relationship between LAPTM4A expression and drug responses but also present novel therapeutic avenues for glioma patients with elevated LAPTM4A expression.

### An FGD5-AS1-hsa-miR-103a-3p-LAPTM4A axis may regulate the progression of glioma

**Figure 10:**
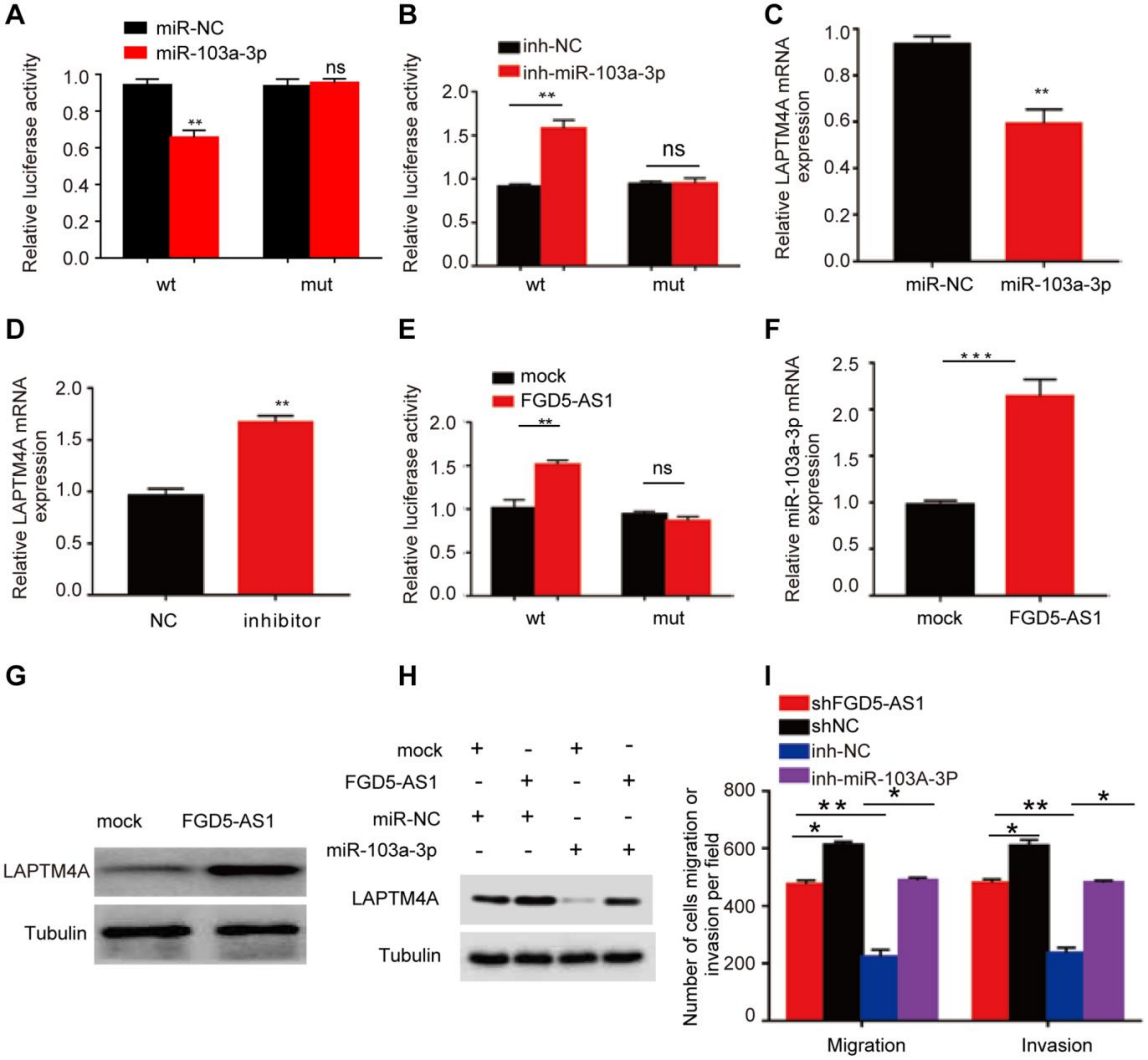
**Prediction of the ceRNA network in glioma.** (**A**, **B**) The regulating relationship of has-miR-103a-3p and LAPTM4A was investigated by a dual-luciferase reporter gene system. (**C**, **D**) Real-time qPCR was used to determine LAPTM4A mRNA levels in U251 cells. (**E**) The regulating relationship of has-miR-103a-3p and FGD5-AS1 was investigated by a dual-luciferase reporter gene system. (**F**) RNA-pull down assay was performed to detect has-miR-103a-3p enrichment in FGD5-AS1. (**G**) Western blotting assay was performed to detect LAPTM4A expression levels in the control and FGD5-AS1 group. (**H**) Western blotting assay was performed to detect LAPTM4A expression levels in the control, FGD5-AS1, and has-miR-103a-3p group. (**I**) Transwell assay was used to detect U251 cell metastasis in the control, FGD5-AS1, inh-NC, and inh-has-miR-103a-3p group. ^*^p < 0.05, ^**^p < 0.01.

Emerging evidence has consistently highlighted the pivotal role of competing endogenous RNA (ceRNA) networks in the context of cancer [46]. In this study, we endeavored to construct a comprehensive ceRNA regulatory network centered around LAPTM4A in gliomas. Through meticulous screening of databases such as TargetScan, DIANAmicroT, and RNAinter, we identified a total of 67 miRNAs that exhibit binding potential with LAPTM4A (Supplementary Figure 9A). Notably, 15 of these miRNAs displayed a negative correlation with LAPTM4A (Supplementary Figure 9B). Given the significance attributed to hsa-miR-103a-3p and hsa-miR-107 in glioma, as elucidated by pertinent literature, we proceeded to investigate the relationship between LAPTM4A expression and these two miRNAs in glioma (Supplementary Figure 9C). Interestingly, a stronger correlation was observed between LAPTM4A and hsa-miR-103a-3p, prompting us to delve deeper into the underlying regulatory mechanism between hsa-miR-103a-3p and LAPTM4A. Intriguingly, putative binding sites were identified, leading to subsequent mutations in LAPTM4A mRNA (Supplementary Figure 9D). Experimental results subsequently revealed that the introduction of the hsa-miR-103a-3p mimic led to a reduction in luciferase activity in U251 cells, while the hsa-miR-103a-3p inhibitor elicited an increase in luciferase activity (Figure 10A, 10B). Moreover, the hsa-miR-103a-3p mimic effectively downregulated LAPTM4A expression in U251 cells, while the hsa-miR-103a-3p inhibitor exerted the opposite effect, elevating LAPTM4A levels (Figure 10C, 10D). Clinically, abnormal down-expression of miR-103a-3p in glioma tissue was observed in comparison to normal adjacent tissue (Supplementary Figure 9E). Furthermore, the expression levels of LAPTM4A in glioma tissue demonstrated a negative correlation with miR-103a-3p (Supplementary Figure 9F).

Subsequently, leveraging the miRNet and starBase databases, we employed predictive analysis to identify potential regulatory relationships between hsa-miR-103a-3p and long non-coding RNAs (lncRNAs) (Supplementary Figure 9G). A scatter plot analysis revealed LAPTM4A exhibited a statistically significant positive correlation with FGD5-AS1 (r = 0.381) (Supplementary Figure 9H). Similarly, FGD5-AS1 as the most significantly correlated lncRNA with miR-103a-3p (r = −0.318) (Supplementary Figure 9I). Encouragingly, potential binding sites between FGD5-AS1 and miR-103a-3p were predicted, further leading to mutations in FGD5-AS1 mRNA (Supplementary Figure 9J). Notably, the dual-luciferase reporter gene system demonstrated that transfection of the FGD5-AS1 mimic successfully reduced relative luciferase activity in U251 cells transfected with wild-type hsa-miR-103a-3p, while no such reduction was observed in cells transfected with the corresponding mutant (Figure 10E). Additionally, over-expressed FGD5-AS1 decreased hsa-miR-103a-3p levels in U251 cells (Figure 10F). Furthermore, overexpression of FGD5-AS1 demonstrated an elevation in LAPTM4A levels in U251 cells (Figure 10G). Strikingly, the co-overexpression of FGD5-AS1 and hsa-miR-103a-3p effectively reversed the heightened expression levels of LAPTM4A (Figure 10H). Notably, Transwell assay results revealed a substantial inhibition in the invasion and migration capacities of U251 cells upon FGD5-AS1 knockdown, which could be rescued by the hsa-miR-103a-3p inhibitor (Figure 10I). Collectively, our findings illuminate the intricate regulatory mechanism governing LAPTM4A, underscoring the significance of the FGD5-AS1-hsa-miR-103a-3p-LAPTM4A axis in the regulation of glioma progression.

## DISCUSSION

Gliomas are the most prevalent and deadliest malignant brain tumor [47], primarily treated through surgery combined with radiation and chemotherapy. However, treatment is often ineffective, leading to frequent recurrence and short survival prognosis. The emergence of molecular biomarkers has had a significant impact on glioblastoma histopathology classification, and diagnosis, as well as on predicting patient survival and treatment response [48]. Previous studies have identified biomarkers such as 1p/19q codeletion [49], isocitrate dehydrogenase [50], EGFR [51], and O^6^-methylguanine-DNA methyltransferase (MGMT) gene promoter methylation [52] as indicators for gliomas. Nonetheless, the routine implementation of these biomarkers in clinical practice has posed challenges, as their association with survival rates and treatment response remains inconclusive, failing to yield substantial clinical benefits for glioma patients [53, 54]. Therefore, the urgent need to identify additional prognostic markers to optimize glioma treatment persists. Through a series of comprehensive and rigorous bioinformatics analyses, complemented by experimental validation, this research establishes LAPTM4A as a novel and potent prognostic factor and therapeutic target for gliomas.

The recent advancements in high-throughput sequencing technology and large-scale cancer genomics databases have enabled a systematic and comprehensive analysis of genes from a machine-learning perspective. In our study, we leveraged the integration of TCGA and GTEx databases and performed WGCNA analysis using differentially expressed genes to identify the modules most relevant to gliomas. Subsequently, through KM survival analysis and the construction of a univariate and multivariate COX regression model, followed by a comparison of clinical significance, LAPTM4A emerged as the most impactful gene.

TCGA combined with GTEx data analysis revealed high LAPTM4A expression in gliomas, GBM, and LGG, and differences in LAPTM4A expression in different clinicopathological groups, including grade, histological type, age, 1p/19q codeletion, IDH status, response to main therapy, and OS events in glioma patients. In addition, we discovered that LAPTM4A in glioma patients had a survival and diagnostic value. We discovered from survival curves that patients with elevated LAPTM4A expression frequently had subpar OS, DSS, and PFI. Both TCGA combined with GTEx data analysis and CGGA data analysis proved this conclusion. Column plots and calibration curves show that LAPTM4A still has good predictive power for patient survival, and ROC curves reveal the diagnostic value of LAPTM4A for glioma.

As the World Health Organization incorporates genetic markers into traditional central nervous system tumor histopathology classification, it underscores the significance of epigenomic alterations in glioma research [55]. Epigenetic mechanisms represent a crucial mechanism of genomic changes, with links to both physiological and pathological events, such as tissue specificity and carcinogenesis [56]. Importantly, gene expression changes are often considered driving factors in carcinogenesis, with genes displaying significant upregulation associated with the hypomethylation of their promoters [57]. Based on these findings, we sought to explain the aberrant overexpression of LAPTM4A in gliomas from the perspectives of methylation status and mutations. Results revealed widespread hypomethylation at the LAPTM4A promoter sites in glioma samples, with differential methylation levels observed across different subtypes, displaying a trend of decreasing methylation levels with increasing glioma grades. On the other hand, inactivation mutations have been shown to modulate the epigenomic landscape [58], working in concert with epigenetic mechanisms to control the process of carcinogenesis. Mutations, when present, synergistically enhance gene expression along with methylation modifications. Studies focusing on the mutation status of LAPTM4A indicate that amplification is the predominant mutation pattern. Taken together, our findings elucidate that the overexpression of LAPTM4A in gliomas can be attributed to both promoter hypomethylation and amplification mutations.

Given that LAPTM4A is a potential prognostic factor in glioblastoma, we are eager to unravel the involvement of LAPTM4A in the biological processes of gliomas. GO suggested that LAPTM4A may be involved in biological processes including neutrophil-mediated immunity, acute inflammatory response, interferon production, regulation of immune effector processes, and humoral immune response. Meanwhile, KEGG pathway enrichment analysis found that LAPTM4A had increased complement and coagulation cascades, phagocytic vesicles, antigen processing and presentation, cytokine-cytokine receptor interactions, and lysosomal pathways. These findings prompted great interest in the role played by LAPTM4A in tumor immunity.

Verhaak et al. identified clinically relevant subtypes of glioblastoma by comprehensive genomic analysis, including classical, mesenchymal, and proneural types, with the mesenchymal subtype characterized by high aggressiveness and resistance to conventional therapy [59]. We found that the sequencing data from Bao, Phillips, and Rembrandt et al. on the Gliovis website suggests that LAPTM4A is highly expressed in the mesenchymal subtype. Moreover, the pathway analysis suggested that LAPTM4A is involved in the EMT process. Not surprisingly, we detected an experimental knockdown of LAPTM4A accompanied by elevated E-cadherin expression and increased expression levels of N-cadherin and MMP9. Further, the Transwell assays confirmed that the shLAPTM4A group inhibited glioma cell invasion and migration compared with the control group. The above results imply that the knockdown of LAPTM4A may restrain the glioma cell metastasis through the EMT process.

The immune microenvironment of gliomas is shaped by factors both extrinsic to tumor cells and intrinsic to tumor cells, operating at multiple levels. In comparison to other peripheral organs, the brain is generally considered “immune-privileged” in immunology [60]. The composition of immune cells in the tumor microenvironment has also been known to impact the prognosis of gliomas [61, 62]. Previous studies have classified tumors as “hot” or “cold” based on their response to immunotherapy. “Hot” tumors exhibit high immune cell infiltration and activated inflammation, while “cold” tumors exhibit the opposite characteristics [63]. Gliomas, in particular, fall under the category of “cold” tumors, characterized by lymphocyte exhaustion and an immunosuppressive tumor microenvironment characterized by T cell dysfunction and abundant immunosuppressive myeloid cells [64–66]. Additionally, Ochocka et al. revealed the functional heterogeneity of gliomas-associated brain macrophages using single-cell RNA sequencing, identifying activation of immunosuppression-related pathways, such as the high expression of CD274 encoding PD-L1, in monocyte/macrophage clusters [67]. The study by Meng et al. [68] highlighted the role of acinar malignant cells in various cancer-related pathways. In our exploration of LAPTM4A expression patterns at the single-cell level in immune cells, we observed high expression in monocyte/macrophage clusters and acinar malignant cell clusters. This suggests that LAPTM4A may regulate the progression of gliomas through immune pathways.

We first elaborated that LAPTM4A expression was closely correlated with the degree of tumor immune infiltration by stromal score, immune score, and estimation score. Moreover, we found that LAPTM4A showed a significant correlation with neutrophil, macrophage, and dendritic cell infiltration in particular. As key components of the tumor microenvironment, macrophages, and cancer-associated fibroblasts provide a supportive stroma for glioma tumor cell expansion and invasion [69–71]. In our study, the TIMER2 website showed a significant positive correlation between LAPTM4A and monocyte/macrophage and cancer-associated fibroblast infiltration. Not only that, our results also reveal a positive correlation between LAPTM4A and multiple immune checkpoint (ICP) gene expressions, including the well-known CD274, PDCD1 [72], and CD80 [73], which tend to suppress effector T cells to promote cancer development [74]. All of the above findings suggest that LAPTM4A may be able to promote glioma progression through immune infiltration and immunosuppression.

Antibody blocking of cytotoxic T-lymphocyte antigen-4 (CTLA-4) or programmed death-1 (PD-1) reduces the inhibition of anti-tumor cytotoxic T-cell responses resulting from the release of negative regulators of immune activation called immune checkpoints (IC) [75]. Whereas not all glioma patients respond well to IC blockade therapy, we attempted to anticipate that patients will respond clinically to immune checkpoint blockade therapy using the TIDE score, with a low TIDE score predicting a better response rate to immunotherapy [76, 77]. Our results revealed that the group with high expression of LAPTM4A had a tendency to have a high TIDE score, implying that overexpression of LAPTM4A may reduce the effectiveness of immunotherapy in glioma patients while lowering LAPTM4A expression may improve the response rate to immunotherapy in patients.

In order to provide effective treatment strategies to glioma patients, we further explored the impact of LAPTM4A expression on drug sensitivity. Through the GSCALite website, we found that patients with over-expression of LAPTM4A were sensitive to nine drugs or small molecules and resistant to 75 drugs or small molecules. Interestingly, in combination with the CTD database, doxorubicin, which reduces LAPTM4A expression, has better efficacy in glioma patients with high LAPTM4A expression. It has been shown that doxorubicin is currently recognized as an effective anticancer agent for glioma treatment [77], and our research supports the administration of doxorubicin to patients with elevated LAPTM4A expression.

The competitive endogenous RNA (ceRNA) network composed of lncRNAs, miRNAs, and mRNA is essential for the regulation of glioma progression [78, 79]. To unveil the potential ceRNA network of LAPTM4A, we initiated our investigation by utilizing three databases to identify miRNAs that show association with LAPTM4A. Notably, He et al.’s study elucidated the significant role of miR-103a-3p in regulating angiogenesis in glioma [80]. Considering the favorable correlation between LAPTM4A and miR-103a-3p, we further delved into the regulatory relationship between them. Our findings confirmed that the miR-103a-3p mimic suppressed LAPTM4A expression in U251 cells, while the miR-103a-3p inhibitor increased the expression levels of LAPTM4A. Additionally, we observed a negative correlation between the expression levels of LAPTM4A and miR-103a-3p, indicating LAPTM4A as a downstream target of miR-103a-3p. Next, we employed two databases to explore the lncRNAs associated with miR-103a-3p. Consistently, FGD5-AS1 exhibited a significant correlation with miR-103a-3p. Previous studies have also demonstrated that FGD5-AS1 activates the Wnt/β-catenin signaling pathway by regulating the miR-129-5p/HNRNPK axis, thus promoting the progression of glioblastoma [81]. With this in mind, we hypothesized the existence of an FGD5-AS1-hsa-miR-103a-3p-LAPTM4A axis in regulating glioma progression. Subsequent experimental validations supported our hypothesis. Initially, we observed the enrichment of miR-103a-3p in the FGD5-AS1 group. Furthermore, overexpression of FGD5-AS1 elevated the levels of LAPTM4A in U251 cells. Moreover, co-overexpression of FGD5-AS1 and hsa-miR-103a-3p reversed the elevated expression levels of LAPTM4A. Finally, we investigated the functional implications of the FGD5-AS1-hsa-miR-103a-3p-LAPTM4A axis. Knockdown of FGD5-AS1 successfully inhibited the invasion and migration of U251 cells, which could be rescued by the hsa-miR-103a-3p inhibitor. Taken together, our findings provide evidence for the involvement of the FGD5-AS1-hsa-miR-103a-3p-LAPTM4A axis as a regulatory mechanism in glioma progression.

However, it is necessary to acknowledge that the present study exhibits several limitations. Firstly, it predominantly relies on online public databases and computational methods. Additional research endeavors are warranted to ascertain the functionality of LAPTM4A, alongside its association with immune cell infiltration Nonetheless, the integration of machine learning algorithms and certain experimental verification fortify the findings of this investigation. Secondly, specific inhibitors targeting LAPTM4A are still under development, and their clinical significance has yet to be validated. Consequently, supplementary clinical investigations conducted within laboratory settings are imperative to authenticate its involvement in glioma.

In conclusion, we found the high expression and better prognostic and diagnostic value of LAPTM4A in glioma and LGG and GBM subtypes and explored that LAPTM4A most probably promoted glioma progression through EMT or immunosuppression pathways. In addition, the FGD5-AS1-hsa-miR-103a-3p-LAPTM4A axis was established to reveal the potential regulation mechanisms of glioma. Finally, doxorubicin may be used to reduce the expression of LAPTM4A to improve the treatment of glioma patients.

## CONCLUSIONS

We utilized the computational biology method and integrated multi-database to identify the most relevant and clinically relevant gene for glioma, LAPTM4A. The results of our study brought strong evidence that LAPTM4A was aberrantly over-expressed in human glioma tissues, which was related to poor survival, clinicopathological characteristics, and clinical subtypes. Secondly, functional enrichment analysis revealed that LATPM4A plays a role in the development of the immune system and cancer. In particular, *in vitro* experiments suggest that LAPTM4A may affect glioma metastasis through the EMT pathway. Further, our study suggested that LAPTM4A is a potential prognostic biomarker associated with immune infiltration in glioma. Furthermore, the knockdown of LAPTM4A may not only be beneficial for immunotherapy but combined with doxorubicin administration may also bring greater therapeutic benefit to patients with glioma. Ultimately, we found that the FGD5-AS1-hsa-miR-103a-3p-LAPTM4A axis promoted glioma metastasis. However, these conclusions await more effective evidence from prospective studies and multicenter clinical trials.

## Supplementary Materials

Supplementary Figures

Supplementary Table 1

Supplementary Table 2
