# Supplementary material for: Identification of a robust biomarker LAPTM4A for glioma based on comprehensive computational biology and experimental verification
Source: Aging (Albany NY). 2024 Apr 12;16(8):6954–89. doi: 10.18632/aging.205736 (PMC11087115; doi:10.18632/aging.205736)
Supplement: Supplementary Figures [file aging-16-205736-s001.pdf]

SUPPLEMENTARY FIGURES

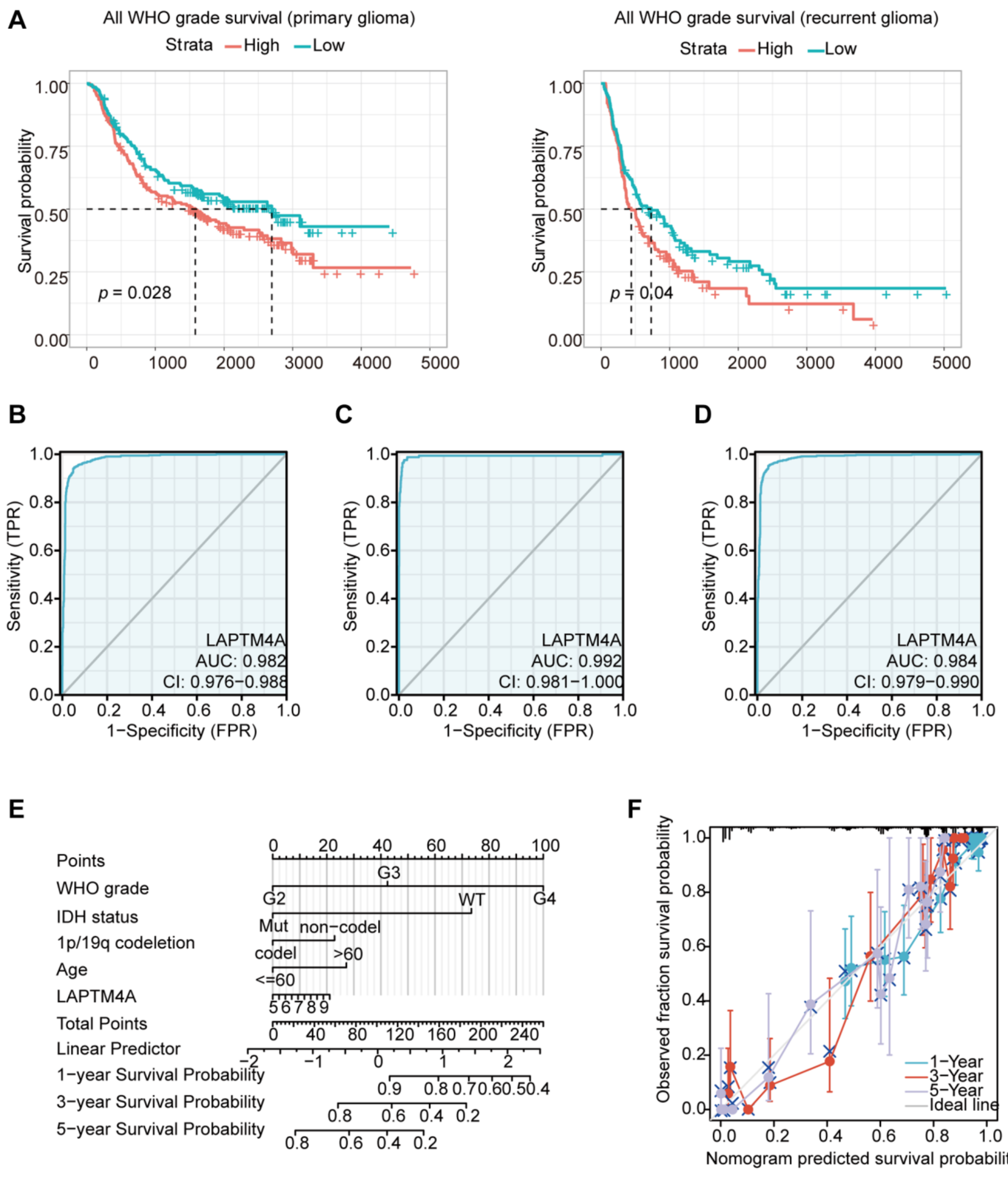

Supplementary Figure 1. Diagnostic significance of LAPTM4A. (**A**) The KM curve of LAPTM4A in the CGGA database revealed that its high expression resulted in a poor prognosis classified by incidence. The diagnostic significance of the LAPTM4A. (**B**) LGG, (**C**) GBM, (**D**) GBMLGG. (**E**) Establishing a nomogram to predict survival in glioma patients. (**F**) The calibration plot of the nomogram of LAPTM4A, showing that the nomogram had good predictive power.

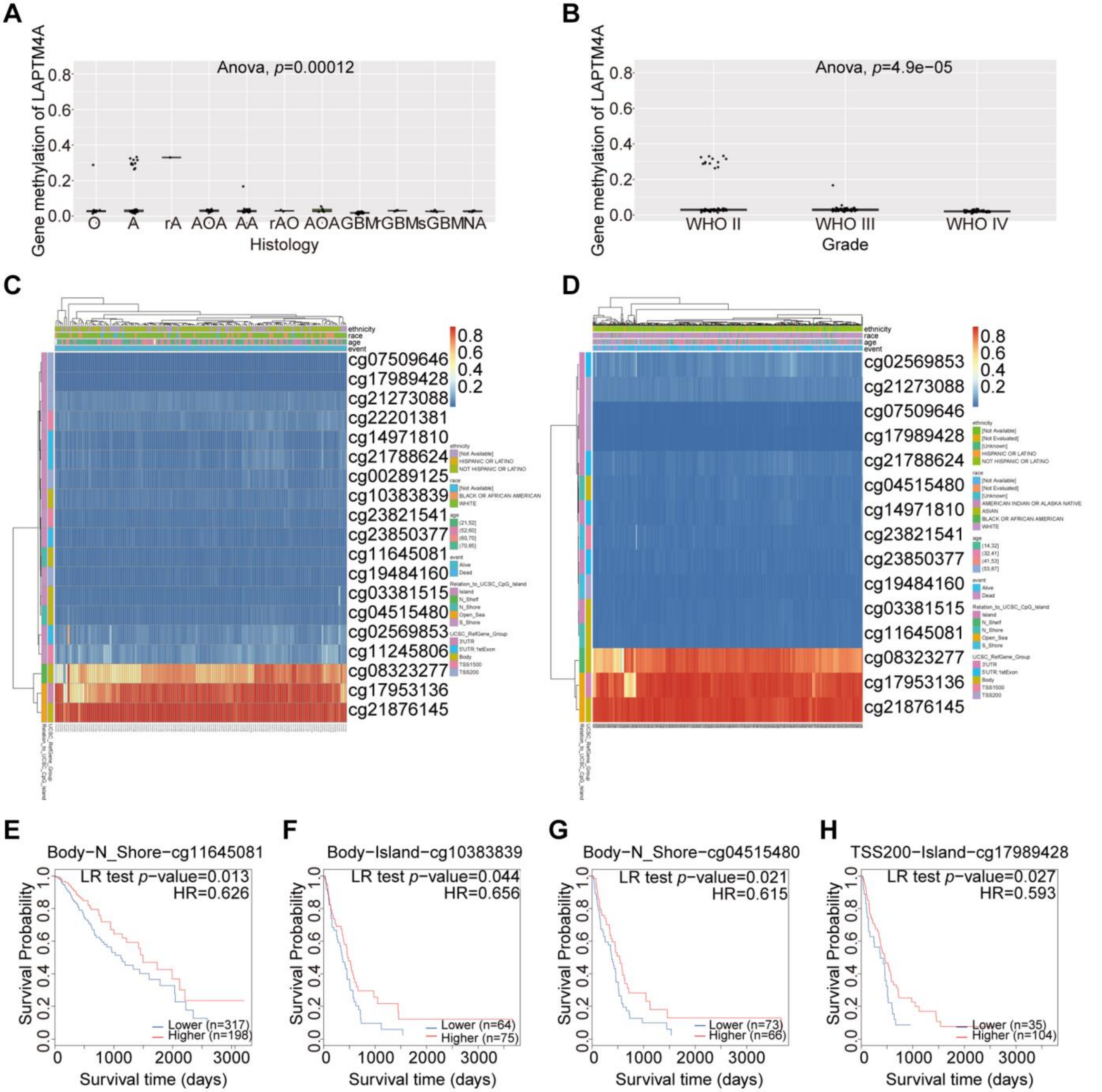

Supplementary Figure 2. Correlation between LAPTM4A promoter methylation level and prognostic value of DNA methylation in GBMLGG. (**A**) Methylation levels of LAPTM4A in different subtypes. (**B**) LAPTM4A methylation levels in the different grades. (**C**) In GBM, LAPTM4A methylation levels at different sites. (**D**) In LGG, LAPTM4A methylation levels at different sites. $^{*}P < 0.05$; $^{**}P < 0.01$; $^{***}P < 0.001$. High methylation level of (**E**) cg11645081 (**F**) cg10383839 (**G**) cg04515480 and (**H**) cg17989428 correlated with worse OS.

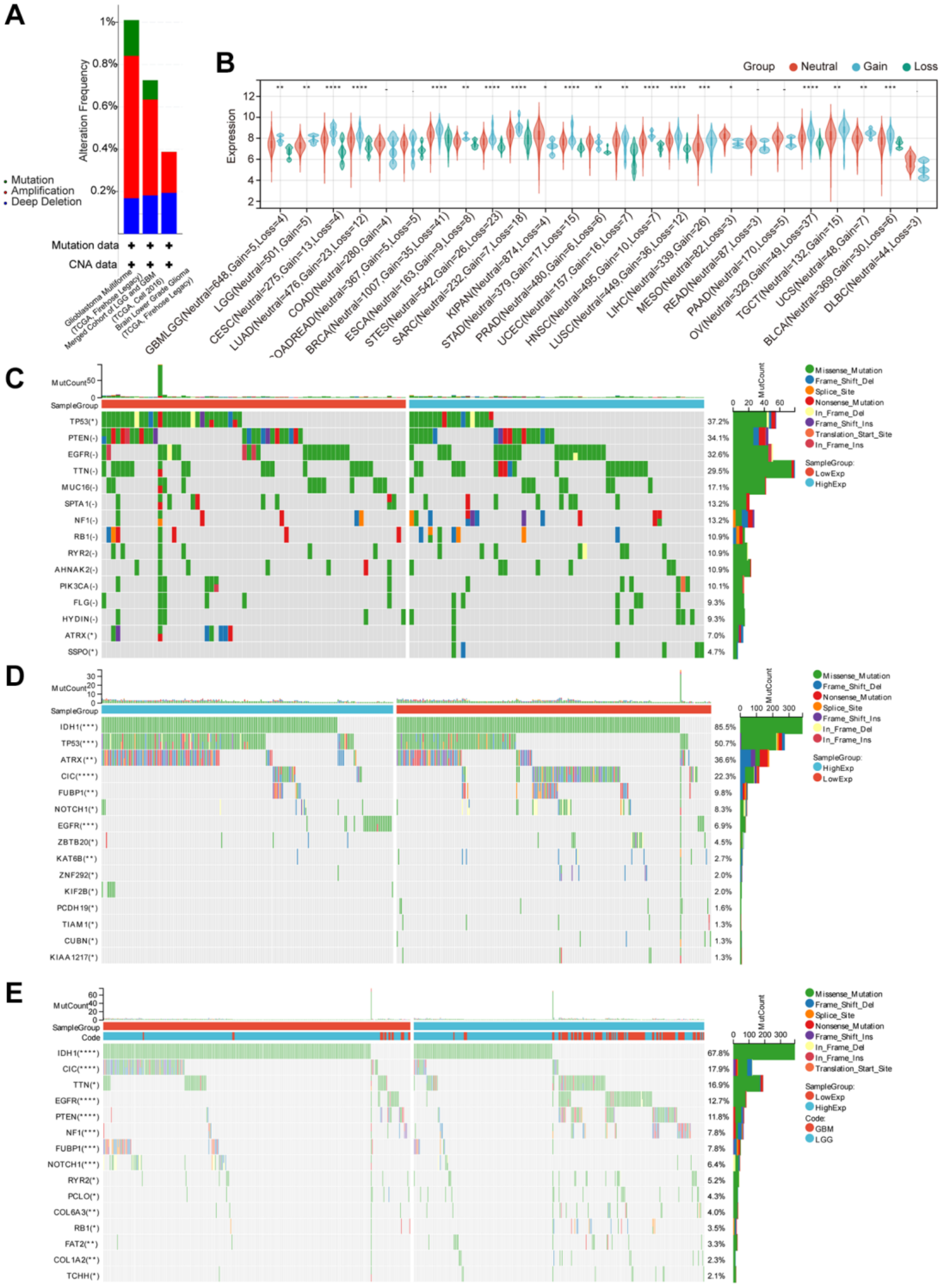

Supplementary Figure 3. Mutational analysis of the LAPTM4A. (**A**) Overall mutation levels of LAPTM4A in gliomas. (**B**) Analysis of copy number variation of LAPTM4A in pan-cancer. LAPTM4A comparison of mutation rates of some functional genes in the high and low expression groups in gliomas. (**C**) GBM, (**D**) LGG, (**E**) GBMLGG.

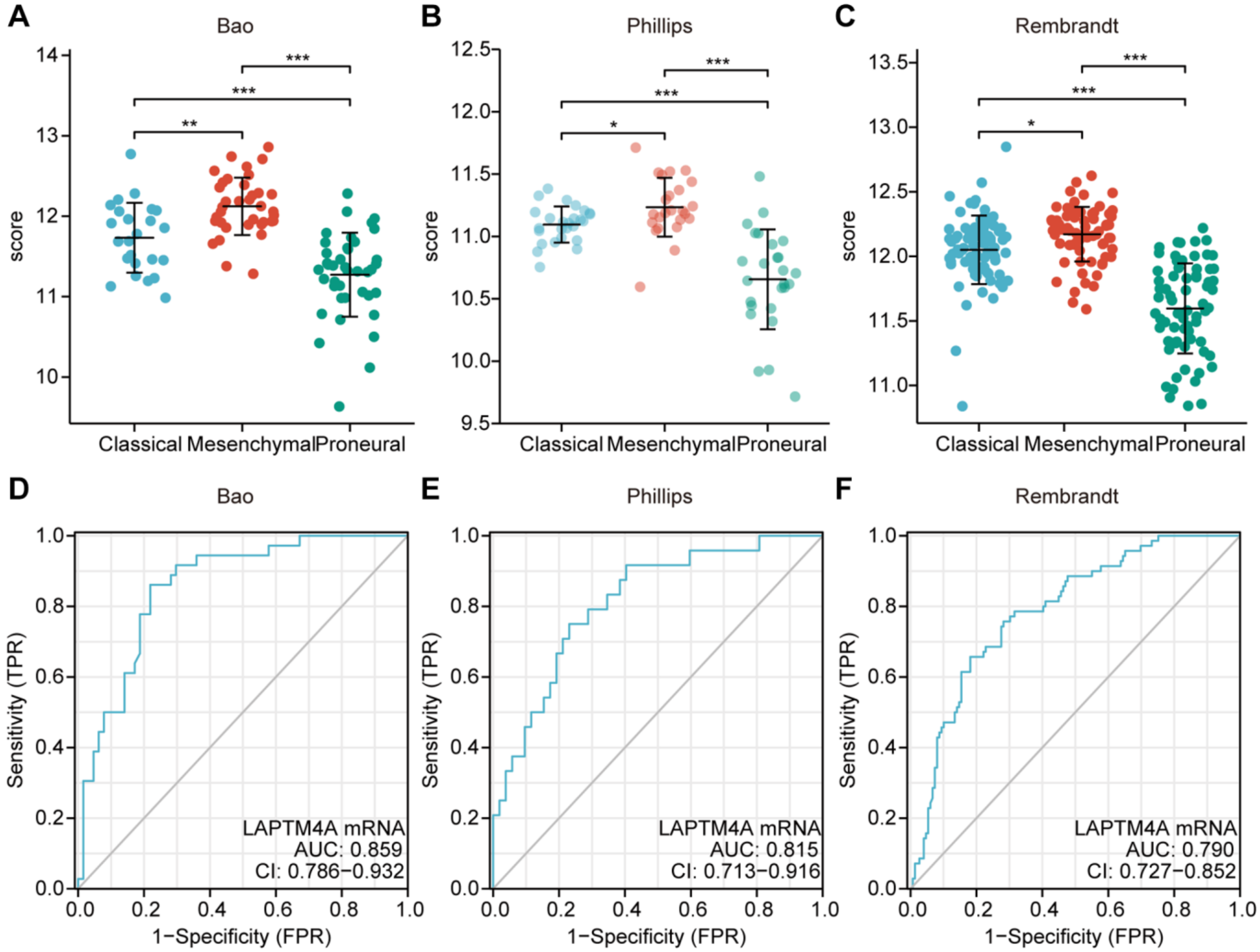

Supplementary Figure 4. LAPTM4A expression enriched in mesenchymal GBM. (**A**–**C**) Three public datasets, namely Bao, Phillips and Rembrandt were used to explore LAPTM4A expression in different molecular subtypes of GBM. (**D**–**F**) ROC curves of LAPTM4A genes in predicting mesenchymal subtype in GBM in Bao, Phillips and Rembrandt. All data were downloaded from Gliovis. Abbreviation: AUC: area under curve. $^{*}p < 0.05$. $^{**}p < 0.01$. $^{***}p < 0.001$. Abbreviation: ns: no significance.

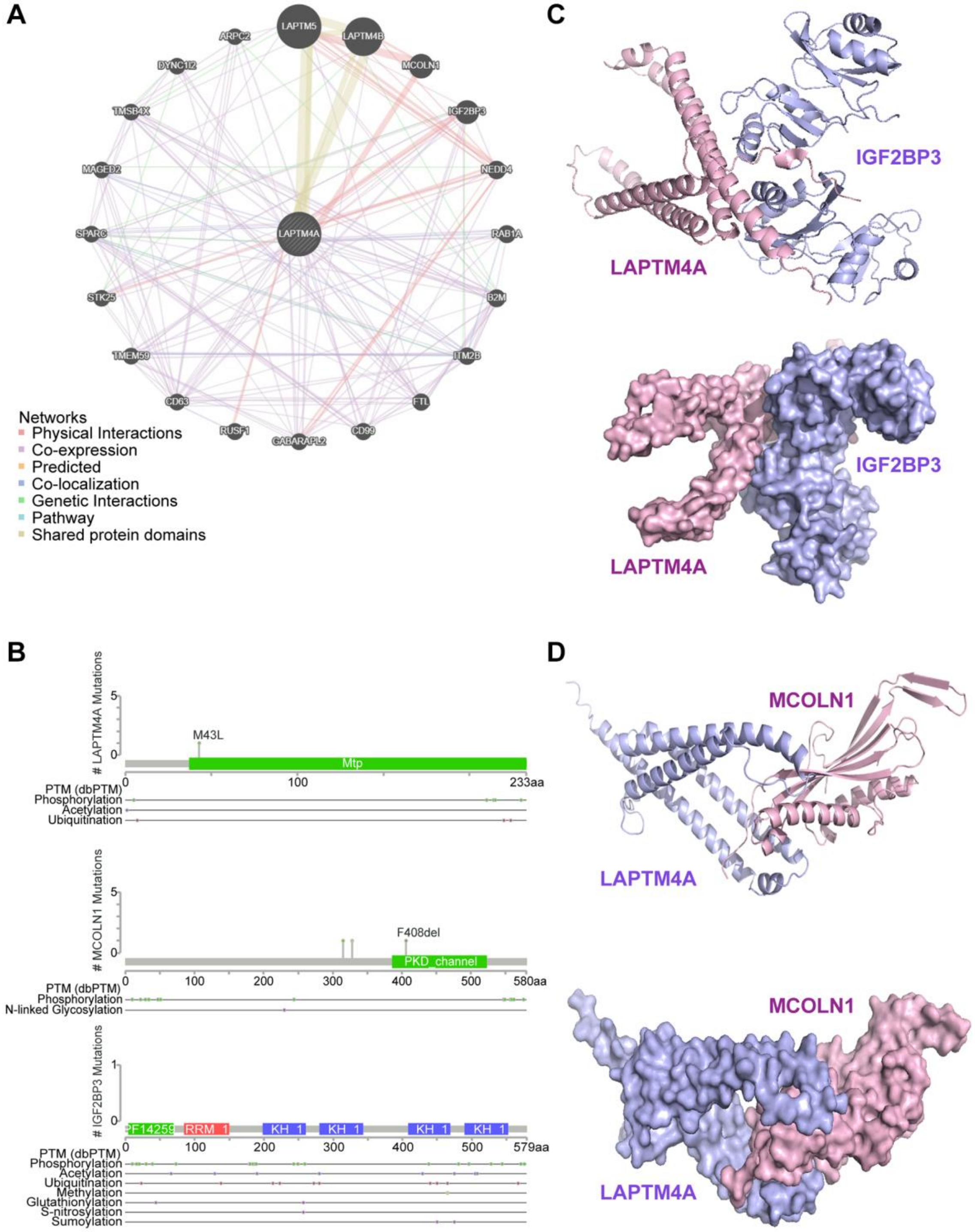

Supplementary Figure 5. PPI network analysis and protein interaction analysis of LAPTM4A. (**A**) The protein interaction network with LAPTM4A were analyzed using the GeneMania website. (**B**) Secondary structure data of LAPTM4A, MCOLN1, and IGF2BP3 downloaded from the cBioPortal database. (**C**) Advanced structure of LAPTM4A versus IGF2BP3, predicting potential binding sites for both. (**D**) Advanced structure of LAPTM4A versus MCOLN1, predicting potential binding sites for both.

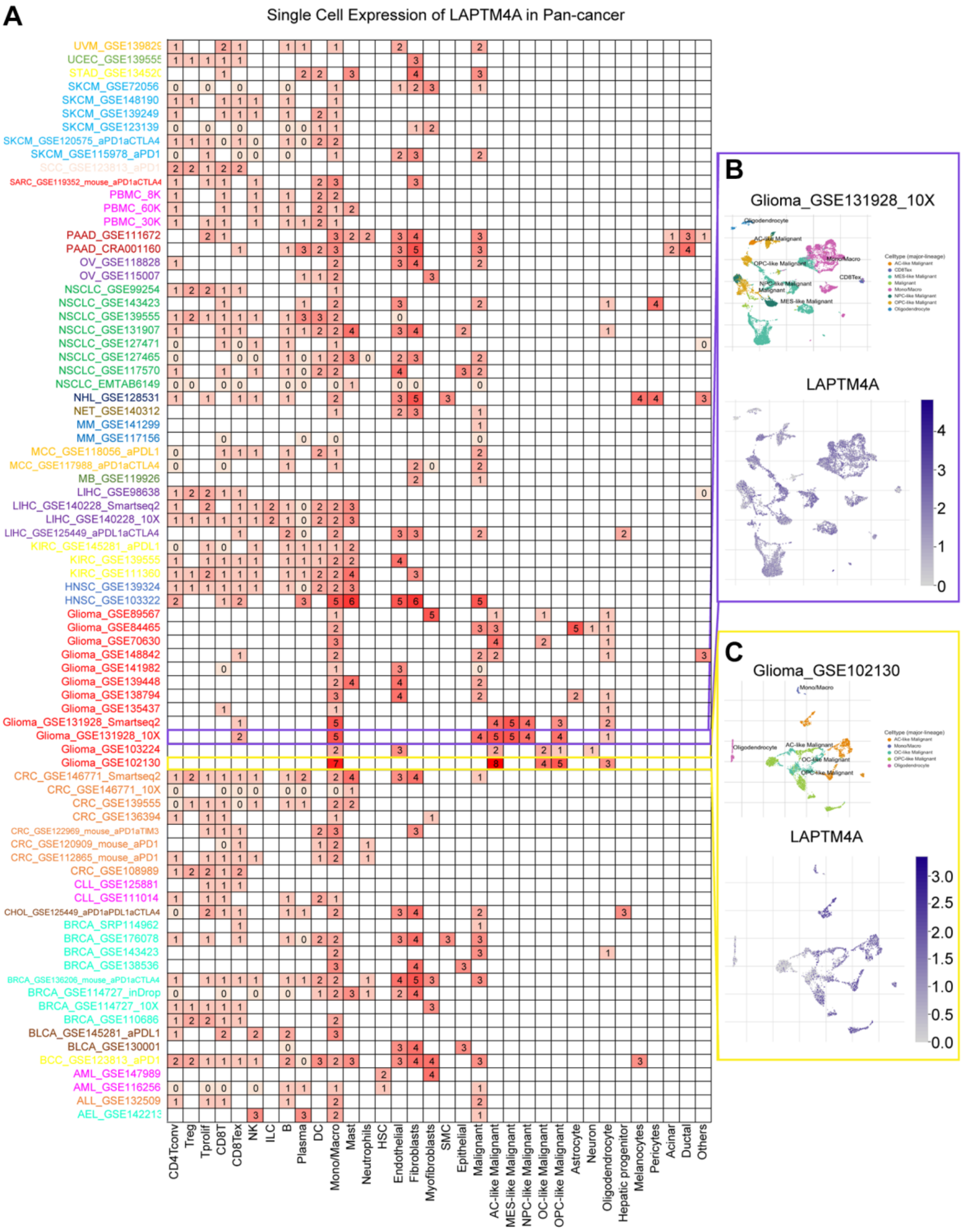

Supplementary Figure 6. The single-cell RNA sequencing analysis exhibits the expression pattern. (**A**) Summary of LAPTM4A expression of 34 cell types in 80 single cell datasets; (**B**) Scatter plot showed the distributions of 8 different cell types for the Glioma_GSE131928_10X dataset. (**C**) Scatter plot showed the distributions of 5 different cell types for the Glioma_GSE102130 dataset.

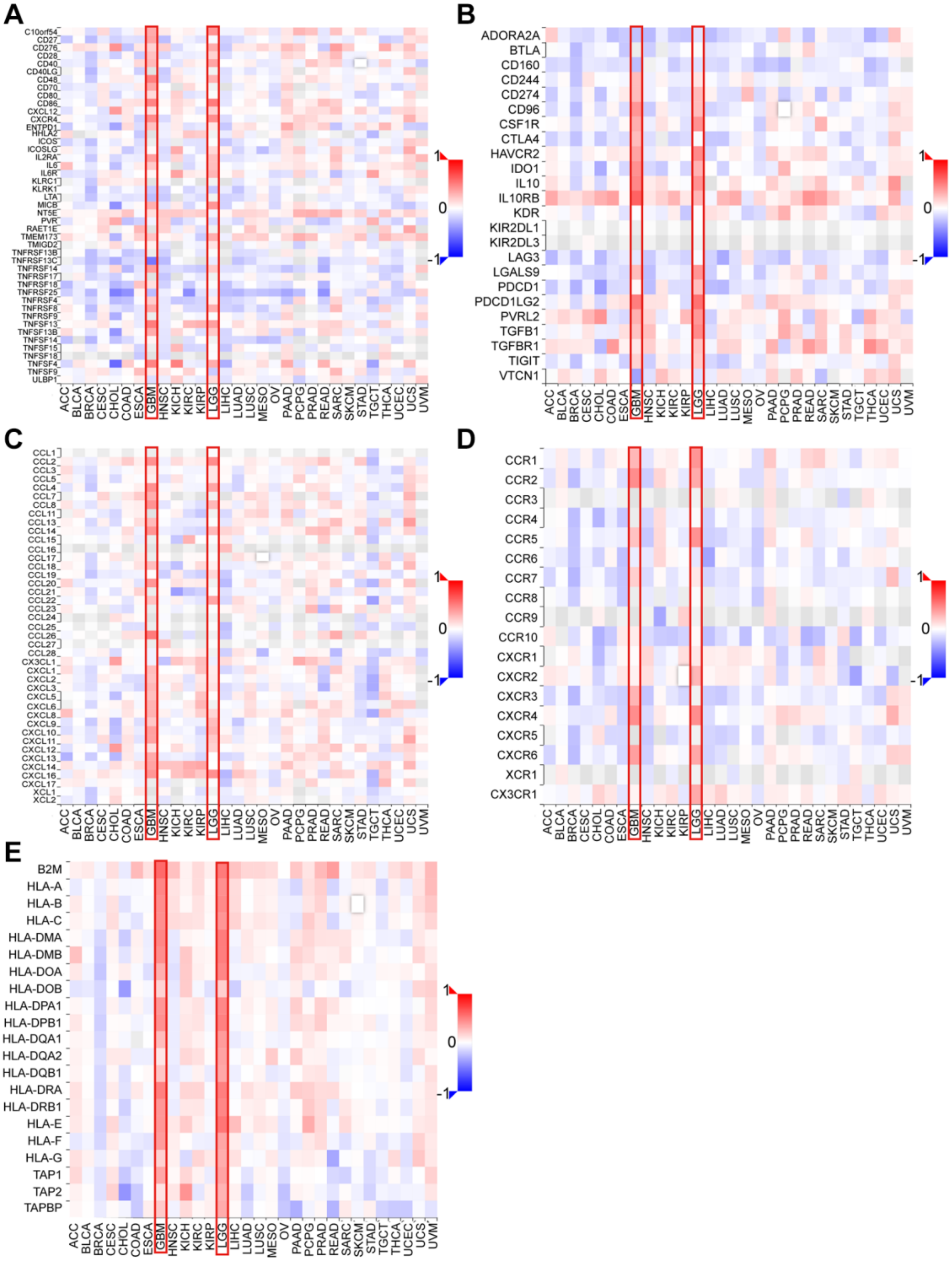

Supplementary Figure 7. Correlation of LAPTM4A with various cytokines in glioma. Correlation of LAPTM4A expression in pan-cancer with (**A**) immunological activation genes, (**B**) immunosuppression genes, (**C**) chemokine receptor (**D**) chemokine receptor, (**E**) MHC molecule.

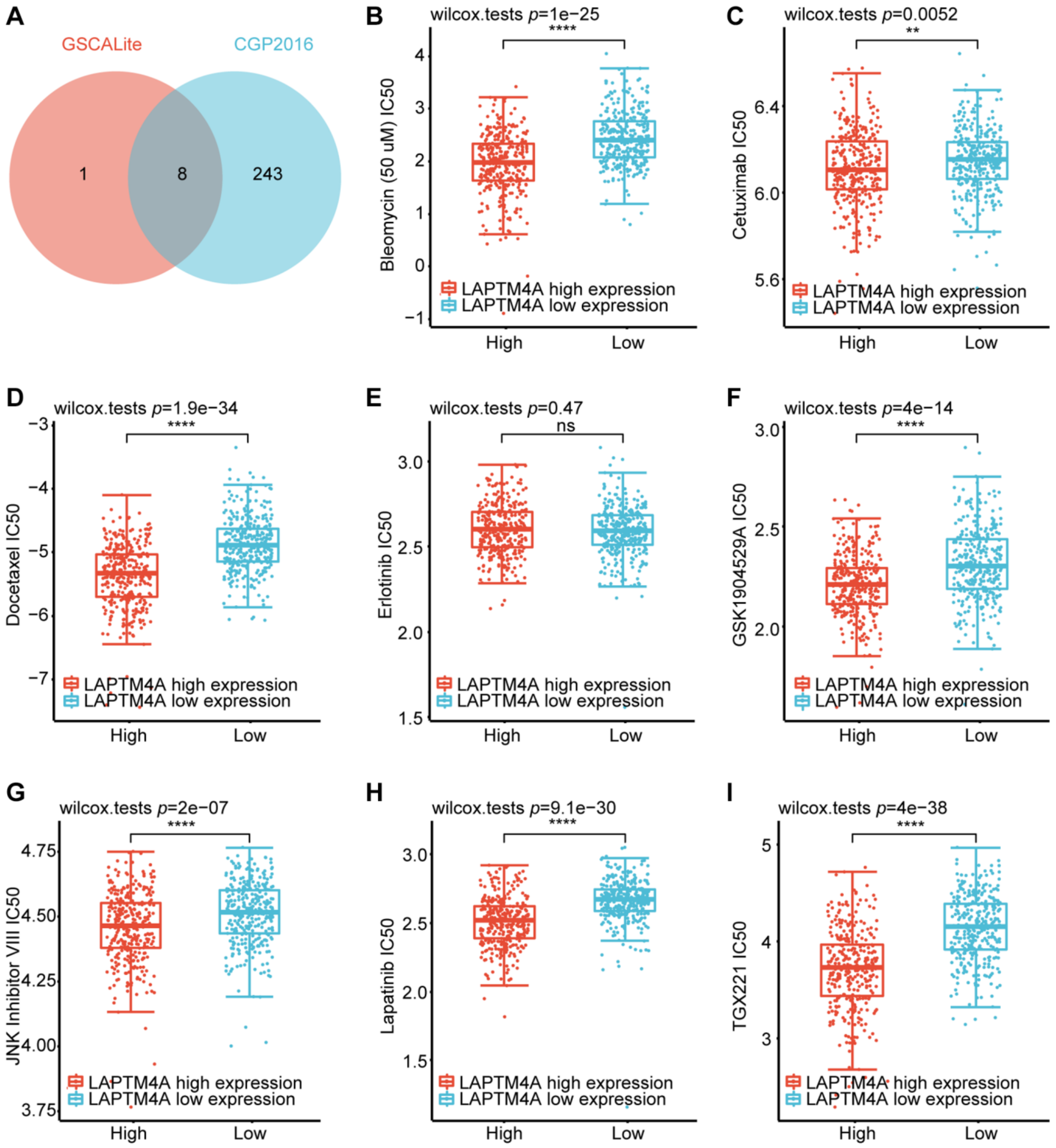

Supplementary Figure 8. Drug sensitivity of LATPM4A in GBMLGG. (**A**) A Venn diagram demonstrates drugs related to LAPTM4A expression in GSCALite and cgp2016. Relationship between LAMP3 expression and IC50 of (**B**) Bleomycin (50 uM), (**C**) Cetuximab, (**D**) Docetaxel, (**E**) Erlotinib, (**F**) GSK1904529A, (**G**) JNK Inhibitor VIII, (**H**) Lapatinib, and (**I**) TGX221.

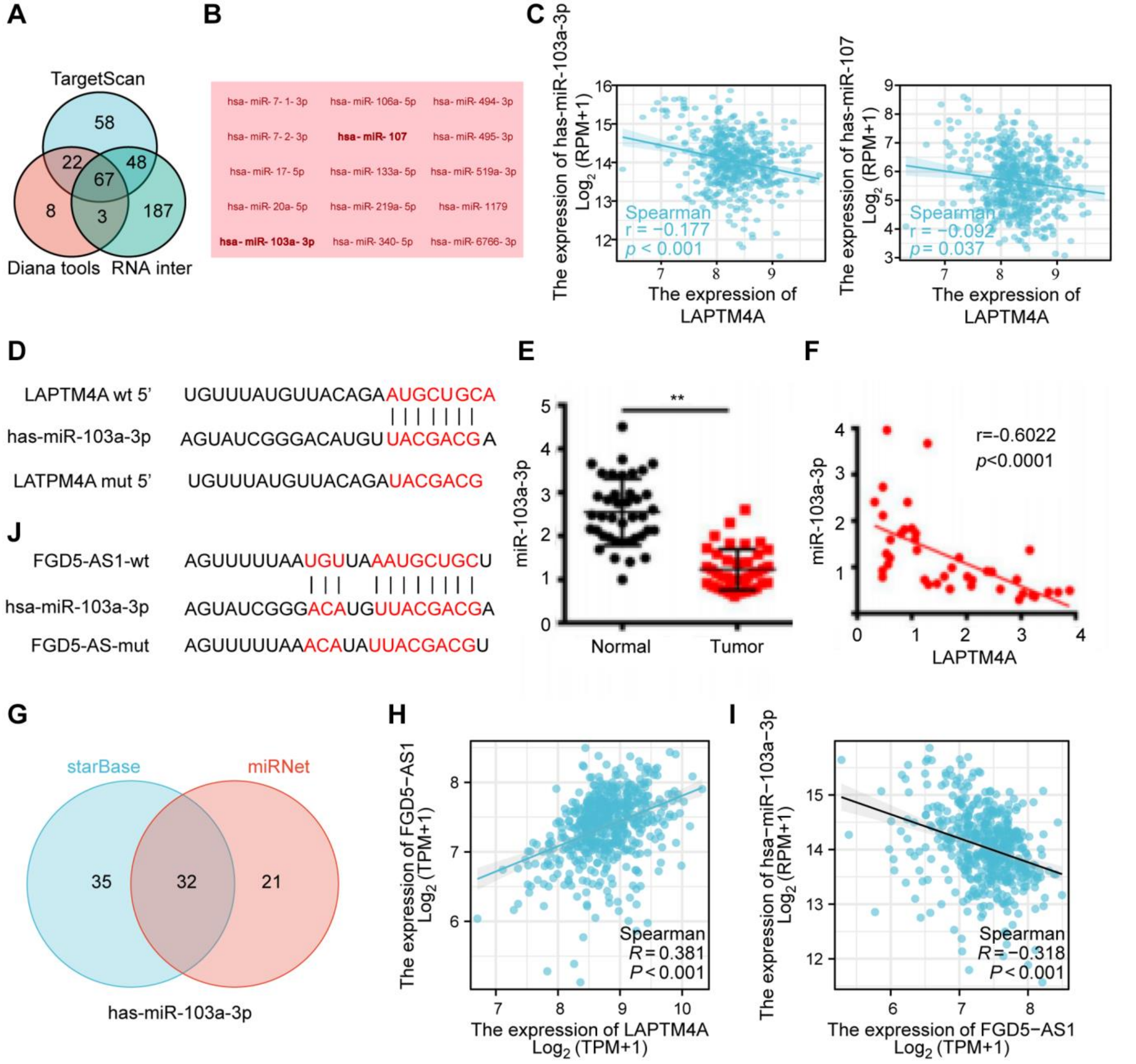

Supplementary Figure 9. ceRNA network analysis of LAPTM4A. (**A**) Venn diagram showing the results for LAPTM4A targets predicted using the TargetScan, DIANA-microT and RNAinter databases. (**B**) 15 miRNA that were negatively correlated with LAPTM4A. (**C**) Scatter plots were generated to show miRNAs-mRNAs with significant correlations. (**D**) Binding sites and mutations of miR-103a-3p and LAPTM4A. (**E**) Real-Time qPCR was used to determine miR-103a-3p mRNA levels in glioma. (**F**) The correlation analysis of miR-103a-3p and LAPTM4A mRNA levels in glioma. (**G**) The lncRNAs that bind to target miRNAs were predicted using the miRNet and starBase online databases and displayed in a Venn diagram. (**H**, **I**) The correlation analysis of LAPTM4A, miR-103a-3p and FGD5-AS1 mRNA levels in glioma. (**J**) Binding sites and mutations of miR-103a-3p and FGD5-AS1. $^{*}p < 0.05$, $^{**}p < 0.01$.
